# EAST Organizes *Drosophila* Insulator Proteins in the Interchromosomal Nuclear Compartment and Modulates CP190 Binding to Chromatin

**DOI:** 10.1371/journal.pone.0140991

**Published:** 2015-10-21

**Authors:** Anton Golovnin, Larisa Melnikova, Igor Shapovalov, Margarita Kostyuchenko, Pavel Georgiev

**Affiliations:** 1 Department of Drosophila Molecular Genetics, Institute of Gene Biology, Russian Academy of Sciences, 34/5 Vavilov St., 119334, Moscow, Russia; 2 Department of the Control of Genetic Processes, Institute of Gene Biology, Russian Academy of Sciences, 34/5 Vavilov St., 119334, Moscow, Russia; Institute of Genetics and Molecular and Cellular Biology, FRANCE

## Abstract

Recent data suggest that insulators organize chromatin architecture in the nucleus. The best studied *Drosophila* insulator proteins, dCTCF (a homolog of the vertebrate insulator protein CTCF) and Su(Hw), are DNA-binding zinc finger proteins. Different isoforms of the BTB-containing protein Mod(mdg4) interact with Su(Hw) and dCTCF. The CP190 protein is a cofactor for the dCTCF and Su(Hw) insulators. CP190 is required for the functional activity of insulator proteins and is involved in the aggregation of the insulator proteins into specific structures named nuclear speckles. Here, we have shown that the nuclear distribution of CP190 is dependent on the level of EAST protein, an essential component of the interchromatin compartment. EAST interacts with CP190 and Mod(mdg4)-67.2 proteins in *vitro* and in *vivo*. Over-expression of EAST in S2 cells leads to an extrusion of the CP190 from the insulator bodies containing Su(Hw), Mod(mdg4)-67.2, and dCTCF. In consistent with the role of the insulator bodies in assembly of protein complexes, EAST over-expression led to a striking decrease of the CP190 binding with the dCTCF and Su(Hw) dependent insulators and promoters. These results suggest that EAST is involved in the regulation of CP190 nuclear localization.

## Introduction

Insulators belong to the class of regulatory elements that organize the architecture of chromatin compartments [1, 2]. Insulators, or chromatin boundaries, are characterized by two properties: they interfere with enhancer–promoter interactions when located between them and buffer transgenes from chromosomal positions effects [3–7]. To date, chromatin insulators have been characterized in a variety of species, which is indicative of their involvement in the global regulation of gene expression. The well-studied *Drosophila* insulator proteins, dCTCF (homolog of vertebrate insulator protein CTCF) and Su(Hw), are DNA-binding zinc finger proteins [8–10].

The Su(Hw) protein, encoded by the *suppressor of Hairy wing* [*su(Hw)*] gene, was one of the first insulator proteins identified in *Drosophila* [9, 11]. The best-studied *Drosophila* insulator found within the 5’-untranslated region of the *gypsy* retrovirus consists of 12 directly repeated copies of Su(Hw) binding sites [8, 12]. Genetic and molecular approaches have led to the identification and characterization of three proteins recruited by Su(Hw) to chromatin—Mod(mdg4)-67.2, CP190, and E(y)2/Sus1—that are required for the activity of the Su(Hw)-dependent insulators [13–17]. The *mod(mdg4)* gene, also known as *E(var)3-93D*, encodes a large set of BTB/POZ protein isoforms [18]. One of these isoforms, Mod(mdg4)-67.2, by its specific C-terminal domain interacts with the enhancer-blocking domain of the Su(Hw) protein [14, 15]. The BTB domain is located at the N-terminus of Mod(mdg4)-67.2 and mediates homo-multimerization [19].

Su(Hw), dCTCF, and most of other identified insulator proteins interact with Centrosomal Protein 190 kD (CP190) [5, 17, 20–25]. This protein (1096 amino acids) contains an N-terminal BTB/POZ domain, an aspartic-acid-rich D-region, four C2H2 zinc finger motifs, and a C-terminal E-rich domain [25, 26]. The BTB domain of CP190 forms stable homodimers that may be involved in protein–protein interactions [19, 25, 27]. In addition to these motifs, CP190 also contains a centrosomal targeting domain (M) responsible for its localization to centrosomes during mitosis [28]. It has been shown that CP190 is recruited to chromatin via its interaction with the DNA insulator proteins in interphase nucleus [17, 24].

The Su(Hw), dCTCF, Mod(mdg4)-67.2, and CP190 proteins colocalize in discrete foci, named insulator bodies, in the *Drosophila* interphase cell nucleus [17, 29, 30]. Contradictory reports have been published in which the insulator bodies are described either as protein-based bodies in the interchromatin compartment [29, 30] or as chromatin domains [17, 22]. As shown recently, insulator proteins rapidly coalesce from diffusely distributed speckles into large punctate insulator bodies in response to osmotic stress [31]. Cell exposure to hypertonic treatment, which enhances molecular crowding, makes it possible to discriminate between nucleoplasmic bodies formed mainly of RNA and proteins (such as PML bodies) and chromatin compartments such as Polycomb bodies formed due to the interaction of distantly located chromatin regions bound by Polycomb proteins [32, 33]. Nucleoplasmic bodies disappear under less crowded conditions and reassemble under normally crowded conditions, which can be interpreted as a consequence of increased intermolecular interactions between components of nucleoplasmic bodies [34, 35]. Similar to PML bodies, insulator bodies are preserved under hypertonic treatment, in contrast to chromatin-based structures that disappear as proteins dissociate from chromatin [35].

The CP190 protein is suggested to be critical for the activity of insulators [26] and to regulate the entry of other insulator proteins into the speckles [30, 31]. At the same time, CP190 associates with centrosomes throughout the nuclear division cycle in syncytial *Drosophila* embryos [36–38]. Nuclear localization of CP190 is also sensitive to various kinds of stress [25, 30, 31], suggesting that this process is highly regulated. However, the mechanisms and proteins responsible for localization of CP190 in different nucleus compartments are unknown.

Here, we have shown that the nuclear distribution of CP190 depends on the level of EAST, which is located mainly in the interchromatin compartment of the nucleus. EAST is a nuclear protein of 2362 amino acids which, except for 9 potential nuclear localization sequences and 12 potential PEST sites, contains no previously characterized motifs or functional domains [39]. Together with Skeletor, Chromator, and Megator proteins, EAST forms the spindle matrix during mitosis [40, 41]. In the interphase nuclei, EAST localizes to the extrachromosomal compartment of the nucleus and is essential for the spatial organization of chromosomes [39, 42–44]. Despite that the bulk of interphase EAST resides in the interchromosomal domain, the current model assumes that EAST can transiently interact with chromosomes [41, 44]. EAST physically interacts with Megator [42], a 260-kDa protein with a large N-terminal coiled-coil domain capable of self-assembly [42]. It has been speculated that Megator can form polymers that, together with EAST, may serve as a structural basis for the nuclear extrachromosomal compartment [41].

Our results show that EAST interacts with CP190 and Mod(mdg4)-67.2 proteins and modulates their aggregation into the nuclear speckles. In case of EAST overexpression, CP190 binding to chromatin is reduced; consequently, the binding of Mod(mdg4)-67.2 and Su(Hw) is reduced as well, since CP190 is essential for it. On the basis of these results, we hypothesize that EAST regulates localization of CP190 and insulator protein complexes in the interchromatin compartment, with these complexes subsequently determining organization of chromatin insulators.

## Materials and Methods

### Construction of plasmids

EAST cDNA was obtained from clone LD33602. The N-terminal part of EAST cDNA (EAST^1-933^) was cloned in pSK by *Xho*I/*Xba*I; the middle part (EAST^933-1995^), in pSK by *Xba*I/*Bam*HI; and the C-terminal part (EAST^1955-2362^), in pGem5 by *Sal*I/*Nde*I. pGAD EAST^1-933^ and pGBT EAST^1-933^ were prepared by ligation of a blunt-ended *Xho*I–*Xba*I fragment from pSK EAST^1-933^ into the corresponding vector by blunt-ended *Eco*RI. pGAD EAST^933-1995^ and pGBT EAST^933-1995^ were prepared by restriction of pSK EAST^933-1995^ with *Xba*I. The resulting fragment was cloned into the pGBT or pGAD vectors digested by *Eco*RI and *Bam*HI. The pGAD EAST^1995-2362^ and pGBT EAST^1995-2362^ plasmids were prepared by cloning the *Nde*I–*Eco*RV EAST^1995-2362^ fragment into pGBT or pGAD digested by *Eco*RI.

To generate EAST^933-2362^ in pGBT and pGAD, the *Bam*HI–*Nde*I fragment obtained from pGem5 EAST^1995-2362^ was cloned into pSK EAST^933-1995^ digested by *Bam*HI and *Sma*I (pSK EAST^933-2362^). The *Xba*I–*Xho*I fragment obtained from pSK EAST^933-2362^ was cloned into either pGBT or pGAD vector digested by *Bam*HI and *Sal*I.

To generate EAST^1-1995^ in pGBT and pGAD, the *Xba*I–*Xho*I fragment from pSK EAST^933-1995^ was cloned into pSK EAST^1-933^ digested by *Xba*I and *Not*I (pSK EAST^1-1995^). Then, the *Xho*I fragment obtained from pSK EAST^1-1995^ was subcloned into either pGBT or pGAD vector digested by *Eco*RI.

For expressing the constructs in S2 cells, we used a modified Invitrogen vector pAc5.1 in which the V5 epitope was substituted by triple FLAG epitope.

To generate pAc EAST^1-933^-FLAG, fragment *Kpn*I–*Not*I from pSK EAST^1-933^ was cloned into the modified pAc5.1 vector digested by the same enzymes.

To generate pAc EAST^933-1995^-FLAG×3, fragment *Not*I–*Xho*I from pSK EAST^933-1995^ was cloned into the modified pAc5.1 vector digested by the same enzymes.

To generate pAc EAST^1995-2362^-FLAG, fragment *Not*I–blunt ended *Nde*I from pGem5 EAST^1995-2362^ was cloned into the modified pAc5.1 vector digested by *Not*I and blunt-ended *Bst*EII.

To generate pAc EAST^1-1995^-FLAG, fragment *Kpn*I–*Xho*I from pSK EAST^1-1995^ was cloned into the modified pAc5.1 vector digested by the same enzymes.

To generate pAc EAST^933-2362^-FLAG, fragment *Xho*I–blunt ended *Xba*I from pSK EAST^933-2362^ was cloned into the modified pAc5.1 vector digested by *Xho*I and *Eco*RV.

Finally, to generate the expression vector for full-length EAST, fragment *Kpn*I–*Bgl*II from pSK EAST^1-1995^ and fragment *Bgl*II–*Nde*I were simultaneously ligated by *Kpn*I and blunt-ended *Bst*EII into the modified pAc5.1 vector.

### S2 cells and RNA interference treatment

The S2 cells cultured as described [45] were transformed using the Effectene Transfection Reagent (Qiagen) or Amaxa Nucleofector kit (Lonza) as recommended by the manufacturer. EAST RNAi was performed with two sets of primers: one specific for the 5’ end of *east* transcript (5’ cgtcggcgaagagatgtcta 3’ and 5’ ttgctctgttactgaggaggatgca 3’) and the other, for the middle part of *east* transcript (5’ taagtcaagcgggaccttgg and 5’ atcccgctgcagaccccat 3’). Both transcripts were used simultaneously to achieve EAST RNAi in S2 cells. An additional EAST RNAi experiment was performed with a second combination of primers specific for the 5’ end of *east* transcript (5'—gaaacccagaatgacaggtgggat- 3' and 5'—gctgttactgttggctccttag- 3').

For CP190 RNAi, we used primers 5’ atgggtgaagtcaagtccg 3’ and 5’ agcgaattccttaacctctt 3’ recognizing the 5’ end of the *cp190* gene. Each primer had a T7 RNA polymerase binding site at 5’ end (5’ ttaatacgactcactatagggaga 3’). The Ambion MEGAscript T7 kit (cat. # 1334) was used to generate dsRNA, which was then heated at 65°C for 30 min and annealed by slowly cooling to room temperature. Drosophila S2 cells were plated in 6-well (35 mm) plates with serum-free medium at a concentration of 1 × 10^6^ cells/mL/well and immediately supplemented with 15 μg dsRNA (5 μL of 3 μg/μL stock solution) per well. The plates were thoroughly swirled and incubated at room temperature for 30–60 min; then 2 mL of the medium with serum was added to each well, and the plates were placed at 27°C. For ChIP analysis and immunostaining assay, cells were treated with dsRNA for 3 days.

### Yeast two-hybrid assay

For growth assays, plasmids were transformed into yeast pJ694A cells (plasmids and protocols from Clontech), which were plated onto media without tryptophan and leucine. After 3 days of growth at 30°C, the cells were plated onto selective media without tryptophan, leucine, histidine, and adenine (high stringency conditions) or without tryptophan, leucine, histidine and 5 mM 3-aminotriazole (medium stringency conditions), and their growth was compared after 2–3 days. The schemes of construct preparation will be provided upon request. For details of the experiment, see S1 and S2 Tables.

### Chromatin preparation and ChIP analysis

The S2 cell suspension was treated with 1% formaldehyde at room temperature for 10 min. The nuclei were washed with PBS and lysed in SDS Lysis Buffer (50 mM Tris-HCl, pH 8.0, with 1% SDS and 10 mM EDTA) by incubation for 20min on ice, and chromatin was sheared by sonication to an average fragment length of about 400 bp. After three rounds of centrifugation, the supernatant was diluted with ten volumes of ChiP Dilution Buffer (16.7 mM Tris-HCl, pH 8.0, with 0.01% SDS, 1.1% Triton X-100, 1.2 mM EDTA, and 167 mM NaCl) and, to reduce nonspecific background, pre-cleared by incubation with protein A or protein G agarose beads for 30 min at 4°C, with constant stirring. Agarose was pelleted by brief centrifugation, and the supernatant was collected for chromatin immunoprecipitation with appropriate antibodies (see below). After overnight incubation at 4°C on a rotary shaker, protein A or protein G agarose beads were added to collect the precipitated complexes, and incubation was continued for 2 h under the same conditions. Agarose was pelleted by centrifugation (700–1000 rpm at 4°C, ~1 min), the supernatant was carefully removed, and the pellet was washed with the following buffers (1 mL each, for 3–5 min on a rotary shaker): Low Salt Wash Buffer (20 mM Tris-HCl, pH 8.0, with 0.1% SDS, 1% Triton X-100, 2 mM EDTA, and 150 mM NaCl), High Salt Wash Buffer (20 mM Tris-HCl, pH 8.0, with 0.1% SDS, 1% Triton X-100, 2 mM EDTA, and 500 mM NaCl), LiCl Wash Buffer (10 mM Tris-HCl, pH 8.0, with 0.25 M LiCl, 1% NP40, 1% deoxycholate, and 1 mM EDTA,), and two portions of TE Buffer. Complex was removed from the agarose by two times elution at room temperature for 15 minutes with rotation with 250 μL of elution buffer (1%SDS, 0.1 M NaHCO3). The eluates were pooled, supplemented with 20 μL of 5 M NaCl, and heated at 65°C for 4 h to reverse the complex–DNA crosslinks. Then 20 μL of 1 M Tris-HCl (pH 6.5), 10 μL of 0.5 M EDTA, and 2 μL of Proteinase K solution (10 mg/mL) were added, and the mixture was incubated for 1 h at 45°C. DNA was recovered by phenol/chloroform extraction and ethanol precipitation and solubilized in water for PCR. PCR products were amplified from three separate immunoprecipitates from at least two different chromatin preparations. Primer sequences used in PCR for ChIP analysis are shown in S3 Table.

### RNA isolation and real-time PCR analysis

For real-time PCR experiments, total RNA was isolated from S2 cells with TRIzol reagent (Invitrogen). Genomic DNA was removed by treatment with DNase I (Fermentas, 1 U per 10 μg) followed by purification with a QIAGEN RNeasy kit. RNA was reverse transcribed into cDNA with a RevertAid H Minus RT Revert Transcriptase (Fermentas) following the manufacturer’s instructions.

The resulting complementary DNA (cDNA) was analyzed by quantitative PCR (Bio-Rad CFX 96 Cycler) using SYBR Green. Relative steady-state mRNA levels were determined from the threshold cycle for amplification by the ΔΔCT method. For each experiment, duplicate or triplicate reactions were performed and averaged, using two independent RNA samples. The expression level of each gene was determined using *ras64B*, *RpL32*, *eh*, and *β-Tubulin56D* as an internal control. Primer sequences used in real-time PCR analysis are listed in S4 Table.

### Antibodies and immunostaining

Specific antibodies and working dilutions were as follows: mouse anti-FLAG (1:300) from Sigma, anti-Tubulin (1:3000) from Abcam, and rabbit anti-Mod(mdg4)-67.2 (1:500), mouse anti- Mod-common (1:500), rat anti-dCTCF C-terminal region (1:300), rat anti-dCTCF N-terminal region (1:300), rat anti-Su(Hw) (1:100) and rabbit anti-Su(Hw) (1:200) raised in our laboratory [29,30]. All experiments were performed with anti-CP190 generated to the C-terminal region of this protein (605–1097 aa) (1:500), except for one additional ChIP experiment with anti-CP190 to its N-terminal region (125–605 aa) (1:500) [30]. The secondary antibodies were Cy3-conjugated anti-rat (Jackson ImmunoResearch), FITC-conjugated anti-rabbit (Jackson ImmunoResearch), and Cy5-conjugated anti-mouse (Jackson ImmunoResearch) IgGs, all used at 1:500 dilution. The S2 cells were grown on coverslips; stained with antibodies against Mod(mdg4)-67.2, FLAG, Su(Hw), or CP190 as described [46]; and examined under a Leica TCS SP2 confocal microscope.

Nuclear extracts and immunoprecipitation experiments were performed as described previously [30].

## Results

### EAST regulates colocalization of CP190 with other insulator proteins in nuclear speckles

**Fig 1:**
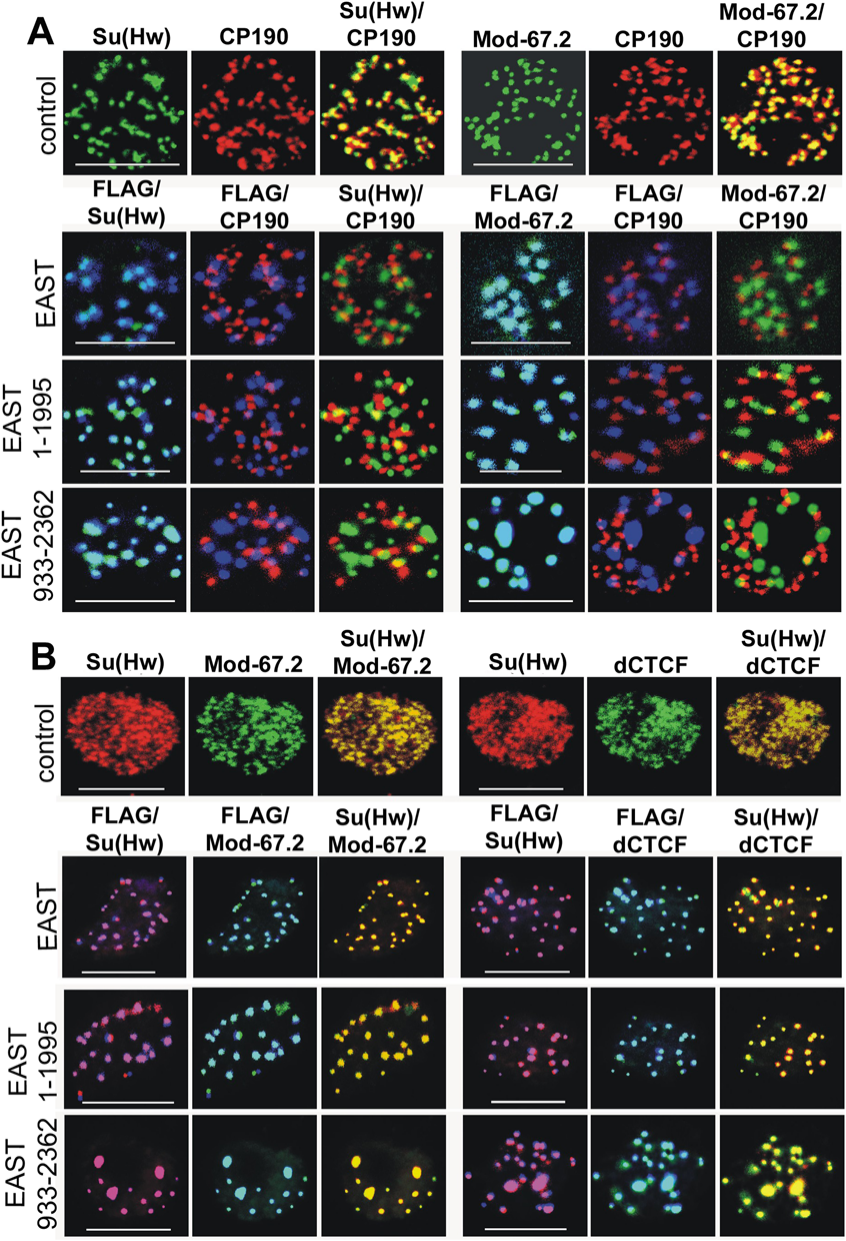
Immunofluorescent localization of insulator proteins in the nuclei of S2 cells overexpressing EAST or its fragments. Immunofluorescent localization of insulator proteins in the nuclei of nontransfected S2 cells (control) and S2 cells overexpressing EAST, EAST^1-1995^, or EAST^933-2362^ tagged with FLAG×3. (A) Immunostaining with antibodies to Su(Hw) (green), Mod(mdg4)-67.2 (Mod-67.2, green), CP190 (red), and FLAG (blue). Scale bars, 5 μm. (B) Immunostaining with antibodies to Su(Hw) (red), Mod(mdg4)-67.2 (Mod-67.2, green), dCTCF C-terminal region (green), and FLAG (blue). Scale bars, 5 μm.

To test whether EAST can influence the distribution of CP190 protein in the interphase nucleus, we examined changes in CP190 localization in the nucleus upon EAST overexpression or inactivation. The nuclei of embryonic *Drosophila* S2 cells contained the speckles or insulator bodies that were positively stained for Mod(mdg4), CP190, and Su(Hw) (Fig 1A and S1 Fig). These speckles were similar in size, number, and distribution to those reported in adult flies [29, 30].

To test the effect of EAST overexpression on the formation of insulator bodies, we compared the distribution of CP190, Mod(mdg4)-67.2, and Su(Hw) insulator proteins in S2 cells transfected with either EAST-FLAG or its truncated variants. EAST tagged with a triple FLAG epitope (EAST-FLAG) under control of the Act5C promoter was transfected into S2 cells. In addition, we expressed two FLAG-tagged partially overlapping truncated forms of EAST, EAST^1-1995^ and EAST^933-2362^. The results of RT-PCR showed that, on average, the amount of *east* transcripts in S2 cells increased tenfold upon their transfection with expression vectors for EAST variants (S2A and S2B Fig). The mRNA encoded by a cDNA gene without introns is usually translated 5–20 times less efficiently than normal mRNA (with introns). Since EAST antibodies were not available for this study, we could not directly estimate the amount of EAST-FLAG protein in transfected cells. Therefore, we used the *su(Hw)* gene, which is expressed in S2 cells at a level comparable to that of the *east* gene. Su(Hw) tagged with a triple FLAG epitope (Su(Hw)-FLAG) under control of the Act5C promoter was transfected into S2 cells (S2C and S2D Fig). The results of RT-PCR showed that the amount of *su(Hw)* transcript in S2 cells increased eightfold upon transfection with the Act5C-Su(Hw)-FLAG expression vector. Using antibodies against the N-terminal domain of Su(Hw), we revealed comparable amounts of Su(Hw)-FLAG and endogenous Su(Hw) in the cells (S2D Fig). Experiments with antibodies against FLAG showed that the amounts of Su(Hw)-FLAG and EAST-FLAG produced in transfected cells were also comparable. Thus, it appeared that FLAG-tagged EAST variants were expressed no stronger than the endogenous EAST protein. If so, the expression of EAST-FLAG in S2 cells resulted in an approximately twofold increase in the amount of EAST protein.

All FLAG-tagged EAST variants colocalized with Su(Hw) and Mod(mdg4)-67.2 proteins in the speckles; on the other hand, CP190 was included in independent speckles. Thus, overexpression of EAST variants proved to reorganize the nuclear speckles in such a way that they contained either CP190 or Mod(mdg4)-67.2 and Su(Hw) together. In addition, overexpression of EAST or EAST^1-1995^-FLAG resulted in an increase in the size of speckles that correlated with a decrease in their number, with overexpression of EAST^933-2362^-FLAG having the most evident effect on speckle size (Fig 1A, S1 and S3 Figs). EAST extensively colocalized with Su(Hw) and Mod(mdg4)-67.2 in the speckles, suggesting that EAST substitutes CP190 in speckle formation. These results suggest that EAST can regulate aggregation of insulator proteins in the nuclear speckles.

It was found that dCTCF in the speckles colocalized with CP190 in the nuclei of imaginal disks. Indeed, the nuclei of S2 cells showed extensive colocalization of dCTCF, Su(Hw), and Mod(mdg4)-67.2, suggesting that dCTCF aggregates with other insulator proteins (Fig 1B and S4 Fig). The overexpression of EAST variants resulted in complete association of dCTCF with Su(Hw) in enlarged speckles, while CP190 was aggregated in independent speckles. Taken together, these results suggest EAST regulates some aspect of nuclear organization that is important for CP190 localization and for integrity of insulators bodies.

**Fig 2:**
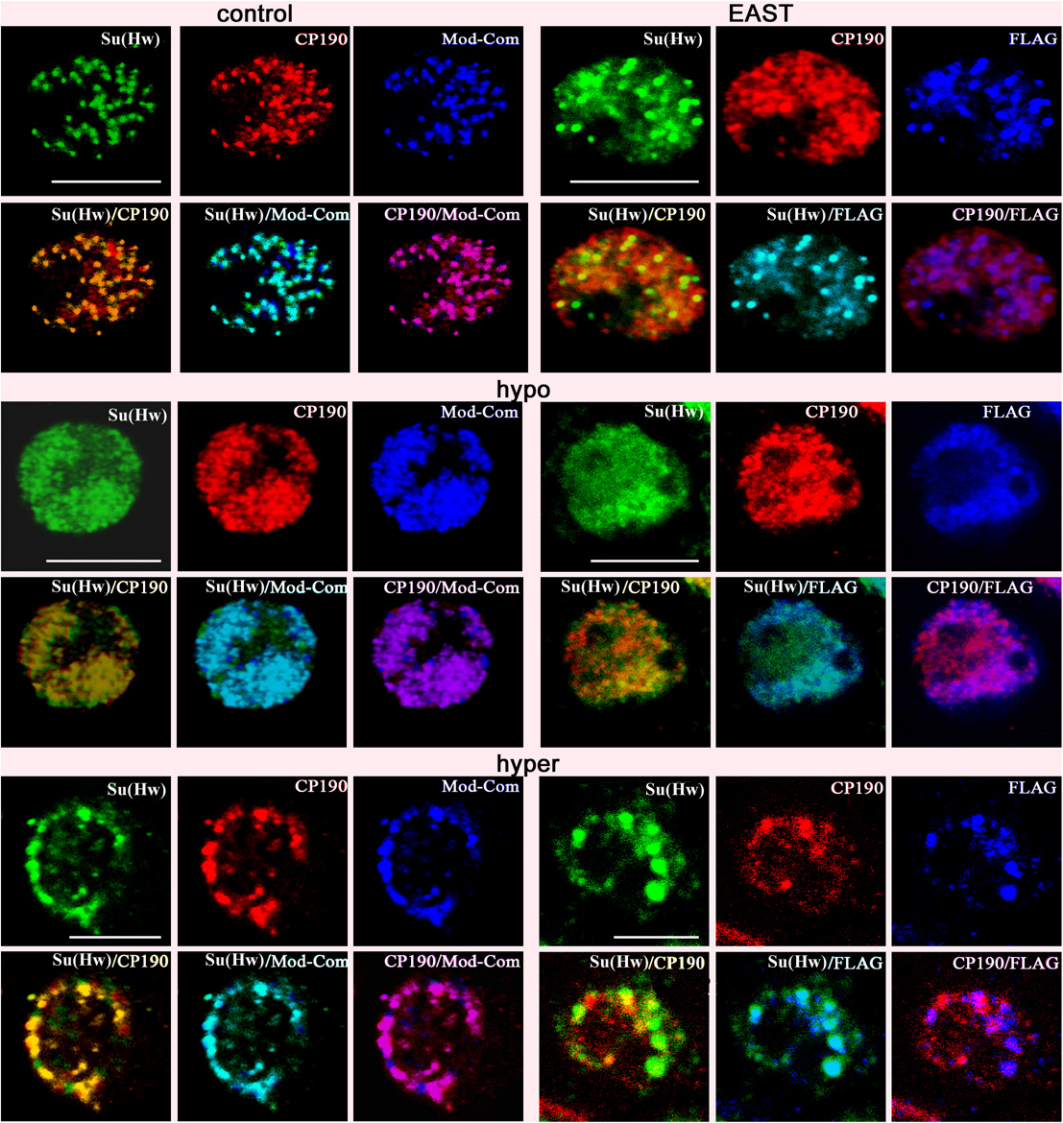
Speckles formed at high and low salt concentrations. Immunofluorescent localization of insulator proteins in the nuclei of nontransfected S2 cells (control) and S2 cells overexpressing EAST^933-2362^ tagged with FLAG×3 (EAST). Immunostaining with antibodies to Su(Hw) (green), CP190 (red), common part of Mod(mdg4) (Mod-Com, blue), and FLAG×3 (blue). Cells were stained in standard SFX medium (isotonic conditions), the same medium diluted fourfold with deionized water (hypotonic conditions), or after treatment with 250 mM NaCl (hypertonic conditions). Scale bars, 5 μm.

As shown previously, the formation of insulator bodies strongly depends on ionic concentration [31]. For this reason, speckle formation in S2 cells was compared under isotonic, hypertonic, and hypotonic conditions (Fig 2). As shown previously [31], osmotic stress induces the formation of larger speckles containing CP190, Mod(mdg4), and Su(Hw) proteins at the periphery of the nuclei, while the size of the speckles formed under hypotonic conditions is reduced. Importantly, in cells expressing FLAG-tagged EAST, we also observed changes in speckle size depending on ionic concentration, but CP190 was still prevalent in independent speckles.

**Fig 3:**
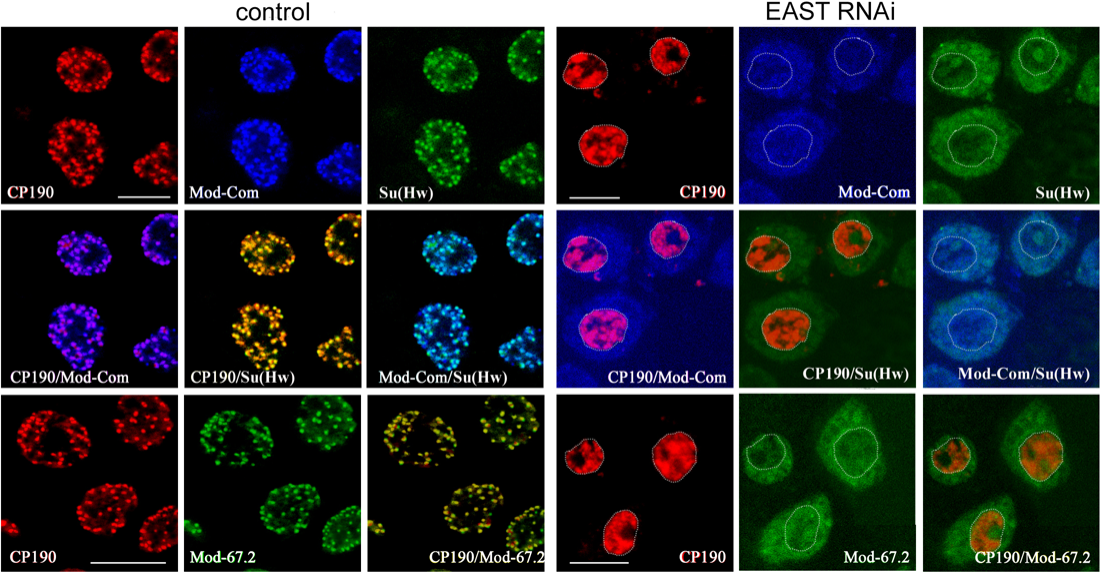
Immunofluorescent localization of insulator proteins in the nuclei of S2 cells after EAST knockdown. EAST RNAi was performed with two sets of primers: one specific for the 5’ end of *east* transcript (5’ cgtcggcgaagagatgtcta 3’ and 5’ ttgctctgttactgaggaggatgca 3’) and the other, for the middle part of *east* transcript (5’ taagtcaagcgggaccttgg and 5’ atcccgctgcagaccccat 3’). Nontransfected S2 cells designated as «control» and S2 cells after EAST knockdown by RNAi designated as «EAST RNAi». Immunostaining with antibodies to CP190 (red), Su(Hw) (green), Mod(mdg4)-67.2 (Mod-67.2, green), and common part of Mod(mdg4) (Mod-Com, blue). Dotted line indicates the nucleus boundary (See S5 Fig). Scale bars, 5 μm.

Next, we examined the formation of insulator bodies after RNAi-mediated knockdown of EAST in S2 cells (Fig 3, S5 and S6 Figs). Strong inactivation of EAST proved to result in a reduction of nucleus size, with CP190 being mainly located inside the nuclei, while Mod(mdg4)-67.2 and Su(Hw) partially colocalizing in the cytoplasm. Thus, EAST is required for the maintenance of Mod(mdg4)-67.2 and Su(Hw) in the nucleus and organization of the proteins in the insulator bodies.

### EAST directly interacts with the insulator proteins CP190 and Mod(mdg4)-67.2

**Fig 4:**
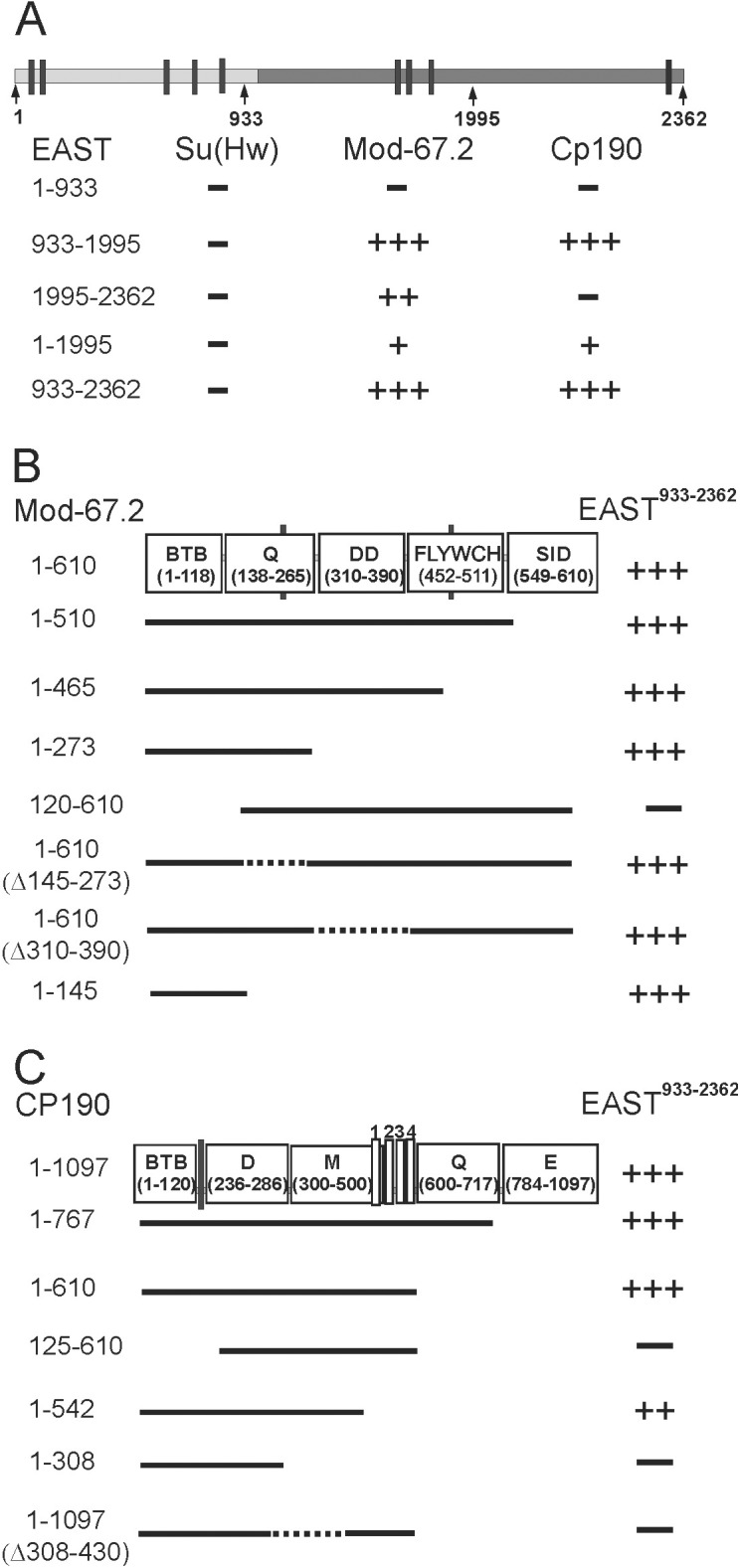
Summary of EAST interactions with insulator proteins in the yeast two-hybrid assay. (A) The results of testing parts of EAST for the interaction with the Su(Hw), CP190 and Mod(mdg4)-67.2 proteins. The scheme shows the structure of full-length EAST, with light and dark gray shading indicating its unstructured and highly structured regions, respectively. For identification of the different EAST protein signatures, InterProScan tool from EMBL-EBI resource was used. It was found that EAST possesses highly structured middle and C terminal regions, in contrast to the unstructured N terminal region. (B) The results of testing Mod(mdg4)-67.2 domains for the interaction with EAST. The scheme shows Mod(mdg4)-67.2 domains with the numbers of amino acid residues included in the corresponding protein product (in parentheses). (C) The results of testing CP190 domains for the interaction with EAST. The scheme shows CP190 domains with the numbers of amino acid residues included in the corresponding protein product (in parentheses), four C2H2 zinc fingers (white rectangles), and the nuclear localization signal (black bar). Figures in the schemes and on the left are the numbers of amino acid residues. The plus signs indicate the relative strength of two-hybrid interaction; the minus sign, the absence of interaction (See S6 Fig).

The strong effect of EAST expression on the formation of insulator bodies may be explained by a direct interaction between insulator proteins and EAST. To test whether EAST can directly interact with components of the Su(Hw) insulator complex, we used the yeast two-hybrid assay (Fig 4 and S7 Fig). The EAST protein (2362 aa) contains 9 sites for potential nuclear localization sequences (NLS) and 12 potential PEST regions that target proteins for rapid degradation [39]. No other motifs have been found within the EAST sequence. To test for a putative interaction between EAST and the insulator proteins, we divided the 2362-aa EAST coding region into three nonoverlapping fragments encoding peptides 1–933 aa (EAST^1-933^), 933–1995 aa (EAST^933-1995^), and 1995–2362 aa (EAST^1995-2362^). Taking into account that the interacting domain could be located at the boundary between two of the above fragments, we also prepared two partially overlapping cDNA fragments, EAST^1-1995^ and EAST^933-2362^.

We performed an interaction study using these fragments of EAST fused to GAL4-activating or DNA-binding domains. The results showed that, the Su(Hw) protein did not interact with parts of EAST (Fig 4A); in contrast, the CP190 protein interacted with the middle EAST^933-1995^ domain, while Mod(mdg4)-67.2 interacted with EAST^933-1995^ and EAST^1995-2362^.

We then examined the domains of CP190 and Mod(mdg4)-67.2 for the interaction with EAST in the yeast two-hybrid assay. The Mod(mdg4)-67.2 protein contains the BTB/POZ domain, glutamine-rich region (Q-rich), dimerization domain (DD), FLYWCH-type Zn finger domain (FLYWCH), and Su(Hw)-interacting domain (SID) (Fig 4B). Using different fragments of Mod(mdg4)-67.2, we found that only its BTB domain was required for the interaction with the EAST^933-2362^ and EAST^1995-2362^ fragments in the yeast two-hybrid assay.

The CP190 protein contains several domains (Fig 4C), including the BTB/POZ domain, aspartic acid rich (D-rich) domain, four C2H2 zinc fingers, and C-terminal glutamic acid rich (E-rich) domain [19]. In addition, it has been shown that a centrosomal targeting domain (M domain) including one zinc finger is responsible for localization of Cp190 to centrosomes during mitosis [18]. In the yeast two-hybrid assay, the interaction of CP190 with the EAST^933-2362^ fragment required BTB, D-rich and M domains (Fig 4C).

**Fig 5:**
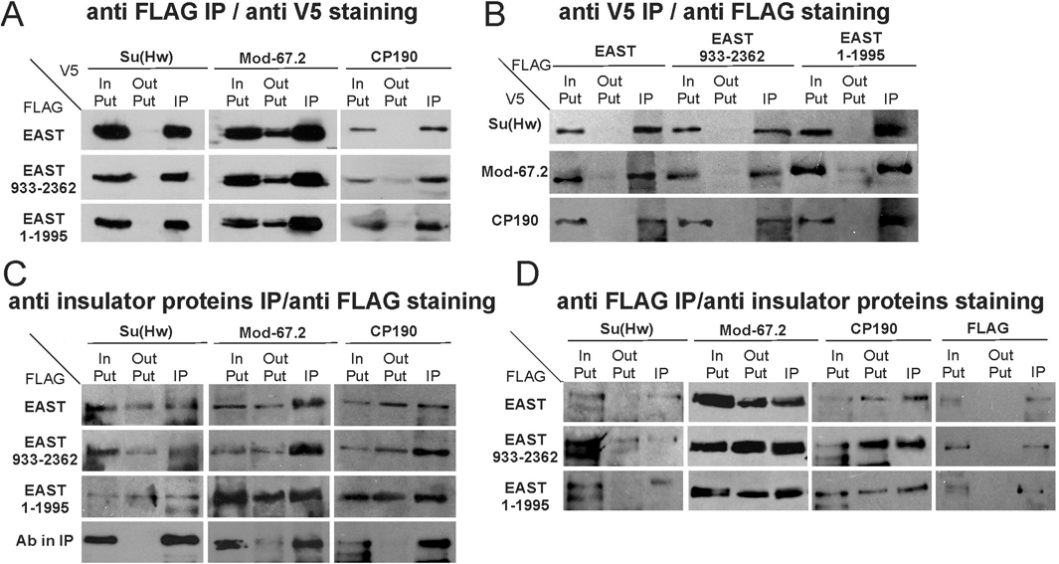
The results of tests for EAST interaction with insulator proteins in S2 cells. (A) Co-immunoprecipitation of V5 epitope-tagged insulator proteins Su(Hw), Mod(mdg4)-67.2 (Mod-67.2), and CP190 by antibodies against the FLAG epitope fused to either EAST or its truncated forms EAST^1-1995^ and EAST^933-2362^. Immunoprecipitated complexes were washed several times with 150 mM KCl-containing buffers and resolved by SDS-PAGE for Western blot analysis with the indicated antibodies. InPut, input fraction (10% of lysate using for immunoprecipitation); OutPut, supernatant after immunoprecipitation; IP, immunoprecipitate. (B) Co-immunoprecipitation of FLAG-tagged EAST variants by antibodies against the V5 epitope fused to either of the insulator proteins. (C) Co-immunoprecipitation between the insulator proteins and the EAST variants fused in frame with FLAG. The S2 cells were transfected with different EAST truncated proteins fused in frame with FLAG. Immunoprecipitation was performed with antibodies raised to insulator proteins Su(Hw), Mod(mdg4)-67.2 (Mod-67.2), or CP190. The bottom panel (Ab in IP) shows the result of immunoprecipitation of target insulator proteins, which was performed in each assay. (D) Co-immunoprecipitation of Su(Hw), Mod(mdg4)-67.2, and CP190 by antibodies against the FLAG epitope fused to each of the EAST variants.

Next, we confirmed the above results by testing the interaction between proteins in S2 cells (Fig 5). Since several attempts to obtain antibodies against different parts of EAST were unsuccessful, we used EAST and its truncated derivatives (EAST^1-1995^ and EAST^933-2362^) tagged with a triple FLAG epitope (EAST-FLAG). EAST-FLAG or its derivatives were coexpressed in S2 cells with each of the insulator proteins tagged with the V5 epitope: Su(Hw)-V5, CP190-V5, or Mod(mdg4)-67.2-V5. As a result, we observed co-immunoprecipitation between the insulator proteins and EAST (Fig 5A and 5B). The co-immunoprecipitation of Su(Hw) and EAST may be explained by the formation of a tight complex between the insulator proteins CP190, Mod(mdg4)-67.2 and Su(Hw). The same results were observed in FLAG-tagged EAST co-immunoprecipitation with endogenous insulator proteins (Fig 5C and 5D). Taken together, these results indicate that both BTB/POZ proteins bound to the Su(Hw) insulator complex effectively interact with EAST.

### EAST overexpression reduces binding of CP190 and Su(Hw)/Mod(mdg4)-67.2 proteins to chromatin

**Fig 6:**
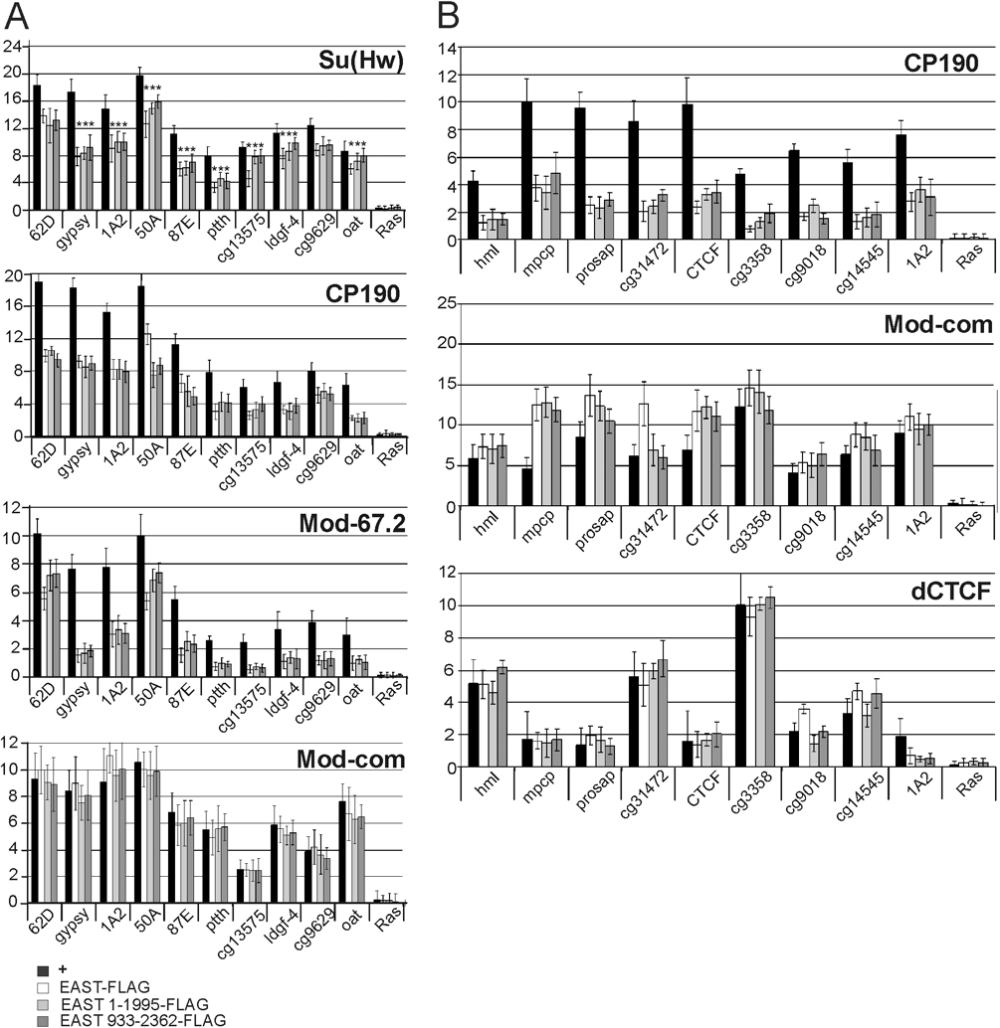
EAST regulates the binding of insulator proteins to their sites in S2 cells. (A) ChIP was performed with antibodies against Su(Hw), CP190, Mod(mdg4)-67.2 (the C-terminal region that corresponds to the specific isoform), and Mod-com (the region common to all Mod(mdg4) isoforms) in normal S2 cells (+) and in transfected S2 cells expressing FLAG×3-tagged EAST, EAST^1-1995^, or EAST^933-2362^. The quantitative PCR (qPCR) was performed on the intergenic and promoter regions bound by Su(Hw). Primers were positioned in the middle of the binding region identified in ModEncode by ChIP-seq. The *ras64B* coding region (Ras) was used as a control devoid of Su(Hw) binding sites. The percent recovery of immunoprecipitated DNA (Y axis) was calculated relative to the amount of input DNA. Error bars indicate standard deviation of four independent biological replicates. *P ≤ 0.05 (Student’s *t*-test), in other cases *P* ≤ 0.01. (B) Results of ChIP with antibodies against CP190, the region common to all Mod(mdg4) isoforms (Mod-com), and dCTCF in normal S2 cells (+) and in transfected S2 cells expressing FLAG×3-tagged EAST, EAST^1-1995^ or EAST^933-2362^. Quantitative qPCR was performed on the promoter regions of eight genes bound to by dCTCF and CP190 and on the 1A2 insulator. Primers were positioned in the middle of the binding region identified in ModEncode by ChIP-seq. Error bars indicate standard deviation of three independent biological replicates.

Next, we examined whether the overexpression of EAST affects the binding of CP190 and other insulator proteins to their sites in S2 cells. We selected genomic sites bound by Su(Hw) in partnership with CP190 and Mod(mdg4)-67.2 [47] and included in analysis the *gypsy* insulator, the 1A2 insulator located on the 3’ side of the *yellow* gene [48, 49], and the intergenic insulators tested previously [50, 51]. In addition, we tested five Su(Hw) binding regions (Fig 6 and S8A Fig) located at distances of less than 500 bp from known gene promoters whose expression was shown to depend on CP190 [20]. We also selected several dCTCF/CP190 binding sites in promoter regions and CP190 binding sites that did not colocalize with known insulator proteins (S8B and S8C Fig).

The *in vivo* binding of the insulator proteins to the Su(Hw) binding sites was examined by ChIP analysis of chromatin isolated from S2 cells transfected with EAST-FLAG, EAST^1-1995^-FLAG, or EAST^933-2362^-FLAG (Fig 6A). As all three EAST variants displayed similar effects, they are hereinafter jointly referred to as EAST overexpression. As shown by RT-PCR, EAST overexpression had no significant influence on the expression of genes encoding insulator proteins: only the transcription of the *cp190* and *su(Hw)* genes was reduced by 15–20% (S9 Fig).

The effect of EAST overexpression manifested itself in twofold reduction of CP190 binding at all tested Su(Hw) binding sites (Fig 6A). It should be noted that similar results were obtained with two variants of antibodies against different parts of CP190, its C-terminal part (605–1097 aa) and N-terminal part (125–605 aa) (data not shown). A similar effect was observed in case of the Mod(mdg4)-67.2 isoform: at some sites such as *gypsy* insulator, its binding was reduced three- to fourfold (Fig 6A). It is noteworthy that all selected regions were enriched to normal levels in ChIP experiments with antibodies against the part of protein sequence common to all Mod(mdg4) isoforms. Thus, the enhancement of EAST expression proved to strongly affect CP190 and lead to Mod(mdg4)-67.2 substitution by other Mod(mdg4) isoforms. However, it is also possible that EAST overexpression leads to partial masking of the specific Mod(mdg4)-67.2 epitope.

EAST overexpression resulted in a decrease of Su(Hw) binding by 10–30%, depending on particular region (Fig 6A). The relatively low magnitude of this decrease might be explained by the observed reduction of Su(Hw) expression by 15–20% in S2 cell transfected with EAST variants. To test this possibility, we examined binding sites that are associated with either Su(Hw) and CP190 (S10A Fig) or Su(Hw) alone (S10B Fig). At all six Su(Hw)/CP190 sites, EAST overexpression resulted in a noticeable (20–40%) decrease of Su(Hw) binding to chromatin (S11A Fig). In contrast, no significant change was observed in Su(Hw) binding to the four sites not bound by CP190 (S11B Fig). These results are consistent with the role of CP190 in the recruitment of Su(Hw) to certain genomic regions.

To test whether or not the negative effect of EAST on CP190 binding to chromatin is restricted to the Su(Hw) sites, we examined eight genomic regions associated with either dCTCF and CP190 or CP190 alone [20, 52]. Interestingly, all eight selected regions proved to be enriched in ChIP experiments with antibodies against the part of the Mod(mdg4) protein common to all its isoforms, suggesting that an as yet unidentified isoform of Mod(mdg4) interacts with dCTCF and CP190 sites (Fig 6B). The overexpression of EAST did not affect the binding of dCTCF or Mod(mdg4) isoforms but significantly reduced the recruitment of CP190 to all test sites (Fig 6B). As shown previously, dCTCF weakly binds to the 1A2 insulator containing only two binding sites for Su(Hw). In this case, EAST overexpression resulted in strong reduction of dCTCF binding to the 1A2 insulator (Fig 6B).

It has been shown that CP190 regulates the binding of Su(Hw) and Mod(mdg4)-67.2 but not of dCTCF and other Mod(mdg4) isoforms [53]. To confirm this observation, we performed experiments with CP190 knockdown in S2 cells (S12 Fig). Indeed, inactivation of CP190 reduced the binding of Su(Hw) and Mod(mdg4)-67.2 but not of dCTCF or other Mod(mdg4) isoforms. These results suggest that EAST overexpression affected chromatin binding by CP190 and other proteins whose recruitment to chromatin depends on CP190.

**Fig 7:**
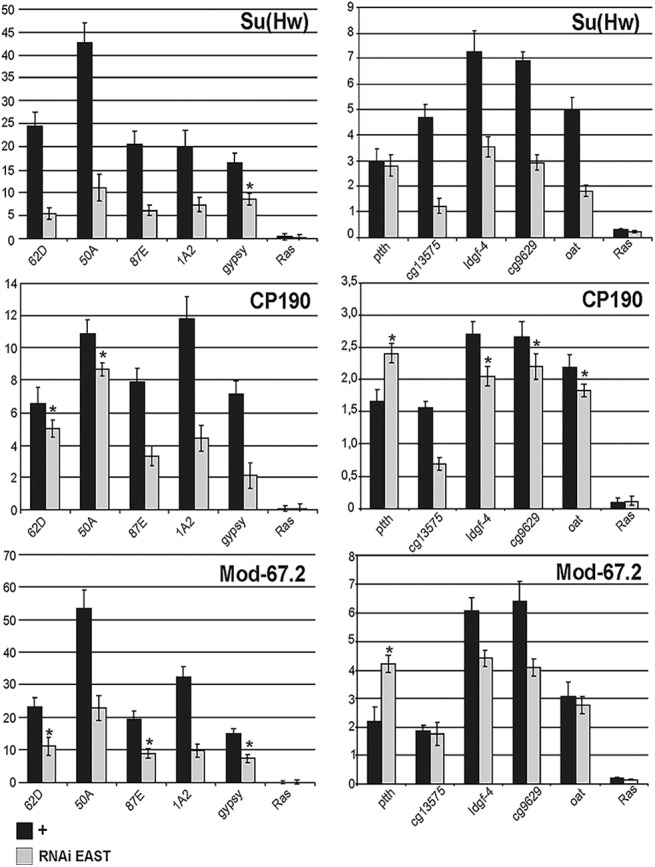
Analysis of insulator proteins binding to chromatin after RNAi knockdown of EAST in S2 cells. ChIP was performed with antibodies against Su(Hw), specific isoform Mod(mdg4)-67.2 (Mod-67.2), and CP190 in normal S2 cells (+) and S2 cells after knockdown of EAST (RNAi EAST). Quantitative qPCR was performed on five Su(Hw)-depended insulator sites and the promoter regions of five genes bound by the Su(Hw) insulator complex. Primers were positioned in the middle of the binding region identified in ModEncode by ChIP-seq. Error bars indicate standard deviation of four independent biological replicates. *P ≤ 0.05 (Student’s *t*-test), in other cases *P* ≤ 0.01. Other designations are as in Fig 6.

Next, we examined the *in vivo* binding of the insulator proteins to the Su(Hw) binding sites in S2 cells after knockdown of EAST by RNAi (Fig 7). The results showed that the binding of the Su(Hw) protein was reduced two- to threefold at eight out of ten sites tested (the *gypsy* insulator and the *ptth* promoter were the exceptions), while that of Mod(mdg4) and CP190 was reduced to a much lesser extent. Interestingly, CP190 and Mod(mdg4)-67.2 binding to the *ptth* promoter was increased in RNAi-treated S2 cells. Thus, inactivation of EAST leads primarily to a decrease in the binding of Su(Hw).

### EAST does not bind to chromatin and has a complex effect on gene expression in S2 cells

**Fig 8:**
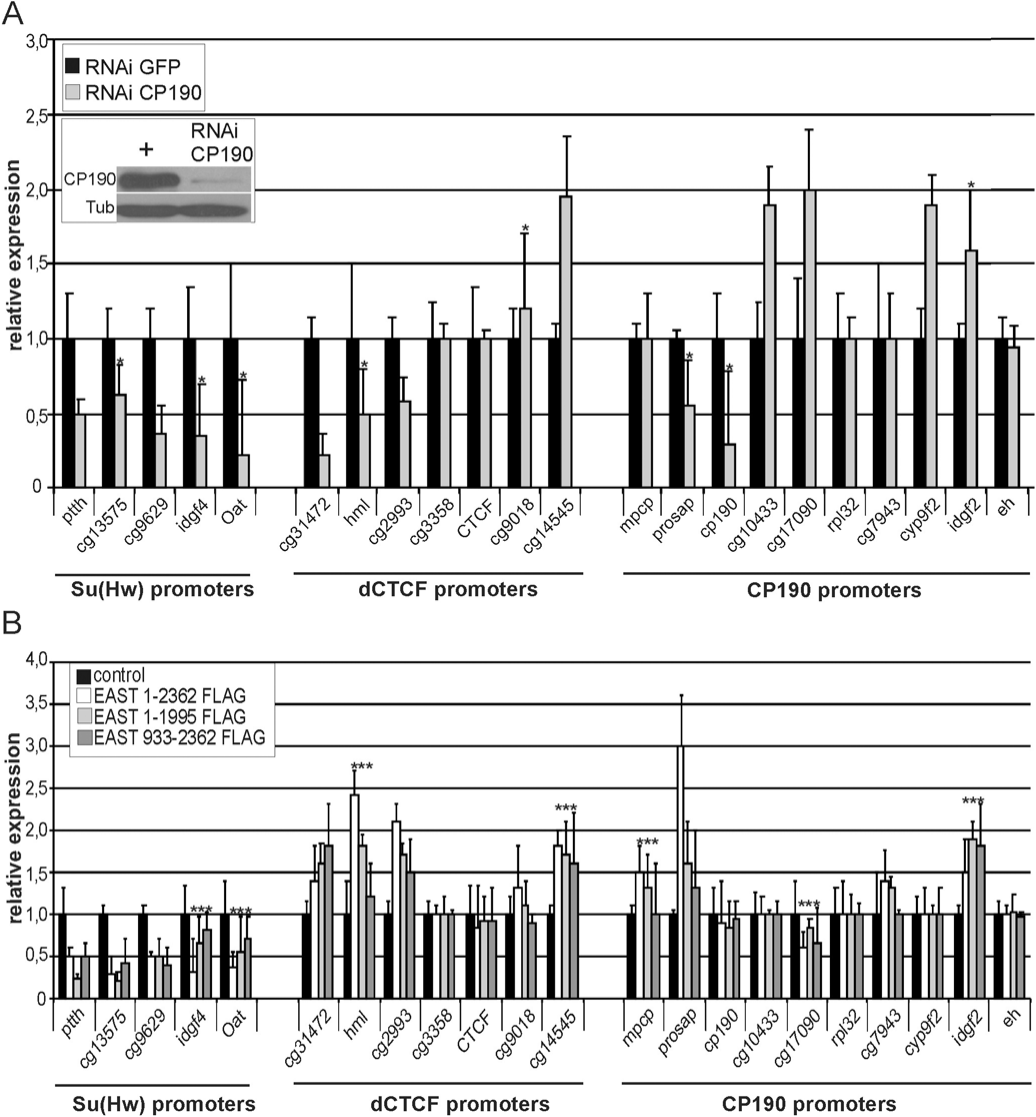
Role of EAST and CP190 in the expression of genes whose promoters are bound to by the insulator proteins. (A) Changes in the expression of individual genes after CP190-specific RNAi knockdown in *Drosophila* S2 cells relative to that after GFP RNAi treatment (taken to be 1). The *RpL32* and *eh* genes that showed no change in expression after the loss of CP190 were used as control. Anti-Tubulin staining (Tub) was used as loading control. The experiments were performed on two samples involving independent RNAi, RNA preparations, and RNA reverse transcription into cDNA. (B) Changes in the expression of individual genes after overexpression of EAST^1-2362^, EAST^1-1995^, or EAST^933-2362^. The experiments were performed on three samples involving independent transfections with a full-length EAST or its fragments, RNA preparations, and RNA reverse transcription into cDNA. Error bars indicate standard deviation of two or three independent biological replicates *P ≤ 0.05 (Student’s *t*-test), in other cases *P* ≤ 0.01.

Therefore, it appears that CP190 inactivation may have the same consequences as enhancement of EAST expression. As shown previously, CP190 knockdown in S2 cells leads to both repressive and activating effects on gene activity [51]. In the case of five promoters strongly bound to by Su(Hw), we observed a twofold reduction of gene expression after either CP190 knockdown or EAST overexpression (Fig 8). In the case of dCTCF-dependent promoters, no correlation in changes of gene expression was found between CP190 knockdown and EAST overexpression: the expression of three out of seven genes was reduced by half after inactivation of CP190 but proved to be enhanced 1.5-fold, on average, upon an increase in EAST expression. Testing additional CP190-dependent promoters also revealed no distinct correlation between EAST overexpression and CP190 inactivation in gene regulation. Thus, high EAST expression has a complex effect on gene expression, which may depend on the combination of the transcription factors bound to the promoter region of the test gene.

According to published data, EAST does not bind to chromosomes under normal conditions [39], but EAST recruitment to polytene chromosomes was observed in permeabilized cells under nonphysiological conditions [44]. We examined whether the overexpressed EAST-FLAG protein could bind to insulator sites. In three replicated experiments, we observed no enrichment in EAST-FLAG on the Su(Hw) or dCTCF binding sites (data not shown). Thus, even overexpression of EAST does not lead to its binding to the insulator sites. However, these results do not exclude close association of EAST with chromatin, which we possibly failed to detect due to limitation of ChIP method.

## Discussion

Our results suggest that insulator bodies are sensitive to the concentration of EAST in interphase cells. The properties of insulator bodies described previously [29, 30] and in this study suggest that they are formed by multiple interactions between proteins and resemble nuclear bodies composed of aggregated proteins and RNAs. As shown previously, the CP190 and Mod(mdg4) proteins interact with Su(Hw) and dCTCF and help the latter to enter the insulator bodies [17, 22, 29, 30]. Taking into account the high level of dCTCF and Mod(mdg4) co-binding to chromosomes, it appears that dCTCF interacts with an as yet unidentified Mod(mdg4) isoform. Mod(mdg4)-67.2 and CP190 conjugate to the small ubiquitin-like modifier protein (SUMO) [30, 54]. Specific interactions mediated by SUMO [29], the ability of Mod(mdg4) BTB to form oligomers [19], and the interaction between the BTB domain of Mod(mdg4)-67.2 and CP190 [46] contribute to specific aggregation of the Su(Hw)/Mod(mdg4)-67.2/CP190 and dCTCF/CP190 complexes into the insulator bodies.

According to current views, the Megator protein can form polymers that, together with EAST, may serve as a structural basis for the nuclear extrachromosomal compartment [41, 42]. The overexpression of EAST leads to an extension of the EAST–Megator compartment, with consequent reduction in the effective volume available for the insulator proteins in the cell. As a result, the concentration of the insulator proteins increases, contributing to stabilization of the compact protein conformations visualized as insulator bodies. By interacting with Mod(mdg4)-67.2 and CP190, EAST may also be directly involved in nucleation of insulator bodies. It is possible that the truncated version of EAST (from 933 to 2362 aa) can more easily interact with the insulator proteins, which leads to noticeable enlargement of insulator bodies in S2 cell expressing EAST^933-2362^.

The overexpression of EAST leads to segregation of the CP190 protein in independent speckles. Our results suggest that EAST interacts with the CP190 region that includes BTB, D, and M domains. These domains are also required for CP190 interactions with other insulator proteins (Golovnin et al., in preparation). Thus, an increase in the EAST concentration may lead to displacement of the insulator proteins from the complex with CP190. Our results do not exclude the possibility that EAST overexpression directly leads to dissociation of CP190 from chromatin. During mitosis, CP190 colocalizes with EAST in the spindle matrix [28, 40, 41], and the increase in the amount of EAST may well be responsible for dissociation of CP190 prior to chromosome condensation.

According to the current model [30], the insulator bodies help to form protein complexes that subsequently bind to regulatory elements such as insulators and promoters. In view of this hypothesis, it is likely that disturbances in the insulator bodies caused by EAST overexpression are responsible for the decrease in CP190 binding to the regulatory regions such as dCTCF- and Su(Hw)-dependent insulators and promoters. As shown recently [53], CP190 is required for recruiting Su(Hw) and Mod(mdg4)-67.2, but not dCTCF, to chromatin. Accordingly, we have observed that EAST overexpression affects the chromosomal binding of Su(Hw), but not of dCTCF. CP190 specifically interacts with the Mod(mdg4)-67.2 isoform [46], and Mod(mdg4)-67.2 at all Su(Hw) binding sites is colocalized with CP190 [53]. Thus, CP190 may be essential for recruiting the specific Mod(mdg4)-67.2 isoform to the Su(Hw) binding sites, with subsequent decrease in the amount of CP190 at the Su(Hw) binding sites, which leads to the substitution of Mod(mdg4)-67.2 by other Mod(mdg4) isoforms, as has been observed in this study.

Strong inactivation of EAST in S2 cells reduces the entry of the Mod(mdg4)-67.2/ Su(Hw) complex, but not of CP190, into the nucleus. It appears that EAST is involved in the regulation of nuclear localization of Mod(mdg4)-67.2, whose BTB domain can form multimeric complexes. Further study is required to elucidate this issue.

## Supporting Information

S1 Fig Immunofluorescent localization of insulator proteins in the nuclei of S2 cells overexpressing EAST or its fragments. S2 cells was transfected full-length EAST, EAST^1-1995^, or EAST^933-2362^ tagged with FLAG×3. Immunostaining with antibodies to Su(Hw) (green), Mod(mdg4)-67.2 (Mod-67.2, green) and CP190 (red). Almost 100% of inspected nuclei show impairment of CP190 co-localization with insulator proteins in speckles. Scale bars, 5 μm.
(TIF)

S2 Fig Expression of the *east* cDNA and its truncated derivatives in the transfected S2 cells. (A) Diagram of lesions in the *east* locus and alternative *east* transcripts. Coding regions and UTRs are shown as black and gray rectangles, respectively; the direction of *east* transcription, by an arrowhead; e1, e2, and e3 are pairs of primers used in qRT-PCR. The numbers of amino acid residues included in the corresponding protein product are shown above the diagram. (B) Expression of the *east* cDNA and its truncated derivatives. Numbers refer to amino acid residues included in corresponding protein products. RNAs were extracted from normal (+) or transfected S2 cells and quantified by RT-qPCR using appropriate primers. mRNA levels were normalized relative to the *ras64B*, *β-Tubulin56D*, and *RpL32* gene expression levels, which remain unchanged in S2 cells transfected with the *east* expression vectors. The experiments were performed on two samples involving independent transfections with a full-length EAST or its fragments, RNA preparations, and RNA reverse transcription into cDNA. Error bars indicate standard deviation of two independent biological replicates. (C) Expression of the *Su(Hw)-FLAG* cDNA. RNAs were extracted from normal (+) or transfected S2 cells and quantified by RT-qPCR using primers Su(Hw) RT fw /Su(Hw) RT rev (S4 Table). mRNA levels were normalized relative to the *ras64B*, *β-Tubulin56D*, and *RpL32* gene expression levels, which remain unchanged in S2 cells transfected with the *Su(Hw)-FLAG* expression vectors. (D) Expression of Su(Hw) protein in S2 cells transfected with EAST-FLAG derivatives. In the top panel, cotransfected EAST-FLAG derivatives and Su(Hw)-FLAG are probed with anti FLAG antibodies. And in bottom panel, the same membrane was probed with anti Su(Hw) antibodies. In the bottom panel we can see that the level of wild type Su(Hw) protein expression is relatively equal to the transfected Su(Hw)-FLAG protein and the presence of EAST expressed derivatives doesn’t significantly influence Su(Hw) expression. Staining with anti-Tubulin (Tub) antibodies (bottom panel) was used as a loading control.
(TIF)

S3 Fig Enlargement of nuclear speckles in cells transfected with full-length EAST or its fragments. Using scale bar as a reference we measured in five nuclei at least twenty speckles diameters. Non transfected nuclei were used as control. Speckles enlargement in cells transfected with full length EAST (1–2362), EAST 1–1995 or EAST 933–2362 represent relatively to the non transfected cells (+).
(TIF)

S4 Fig Immunofluorescent localization of Su(Hw) or Mod(mdg4)-67.2 and dCTCF proteins in the nuclei of S2 cells. Immunostaining with antibodies to Su(Hw) (green), Mod(mdg4)-67.2 (Mod-67.2, green) and dCTCF (red). This staining was done using distinct (N-terminal region) anti-dCTCF (CTCF) antibodies. Scale bars, 5 μm.
(TIF)

S5 Fig Immunofluorescent localization of insulator proteins in the nuclei of S2 cells after EAST knockdown by RNAi. Immunostaining with antibodies to CP190 (red), SuHw (green), and Mod(mdg4)-67.2 (green). DAPI staining (blue) was used to visualize the nuclei. Scale bars, 5 μm.
(TIF)

S6 Fig Immunofluorescent localization of insulator proteins in the nuclei of S2 cells after a EAST knockdown by RNAi with dsRNA generated using a second combination of primers. Nontransfected S2 cells designated as «Control» and S2 cells after EAST knockdown by RNAi designated as «EAST RNAi». Immunostaining with antibodies to CP190 (red), Su(Hw) (green), Mod(mdg4)-67.2 (Mod-67.2, green), and common part of Mod(mdg4) (Mod-Com, blue). Dotted lines indicate the nucleus boundaries (See S5 Fig). Scale bars, 5 μm. The second combination of primers (5'—gaaacccagaatgacaggtgggat- 3' and 5'—gctgttactgttggctccttag- 3') was generated for the 5’ end of *east* transcript.
(TIF)

S7 Fig Test for two-hybrid interaction strength: (1) ++, (2) no interaction, (3) +, (4) +++. (TIF)

S8 Fig Genome browser view of insulator protein binding to (A) Su(Hw) promoters, (B) CP190 promoters, and (C) dCTCF promoters. Genes examined for expression are marked red. The regions used in qPCR are indicated by vertical white arrows and numbered according to their chromosome position (FlyBase, 2006).
(TIF)

S9 Fig Effect of EAST overexpression on the transcription of insulator proteins in S2 cells. (A) Scheme of the *mod(mdg4)* gene encoding the Mod(mdg4)-67.2 isoform. The mRNA for the Mod(mdg4)-67.2 protein is formed by trans-splicing of two RNAs encoded by genes located on the opposite DNA strands. Positions of primer pairs used in qRT-PCR (m1, m2, and m3) are indicated by arrows. (B) Expression of the gene encoding Mod(mdg4)-67.2 in normal S2 cells (+) and in transfected S2 cells expressing FLAG×3-tagged EAST^1-2362^, EAST^1-1995^ or EAST^933-2362^. (C) Expression of *su(Hw)*, *DCTCF* and *cp190* genes in S2 cells transfected with EAST variants. mRNA levels were normalized relative to the level of *ras64B*, *β-Tubulin56D*, and *RpL32* gene expression, which remains unchanged in S2 cells transfected with the *east* expression vectors. The experiments were performed on two samples involving independent transfections with a full-length EAST or its fragments, RNA preparations, and RNA reverse transcription into cDNA. Error bars indicate standard deviation of two independent biological replicates.
(TIF)

S10 Fig Genome browser view of insulator protein binding to (A) regions bound by Su(Hw) and CP190, and (B) regions bound by Su(Hw) alone. The regions used in qPCR are indicated by vertical black arrows and numbered according to their chromosome position (FlyBase, 2006). Names of the identified genomic regions are given in the upper right corner of pictures.
(TIF)

S11 Fig EAST overexpression affects binding of insulator proteins to their sites in S2 cells. ChIP was performed with antibodies against Su(Hw), CP190 and Mod(mdg4)-67.2 (the C-terminal region that corresponds to the specific isoform) in normal S2 cells (+) and in transfected S2 cells expressing FLAG×3-tagged EAST, EAST^1-1995^, or EAST^933-2362^. The *ras64B* coding region (Ras) was used as a control devoid of Su(Hw) binding sites. The percent recovery of immunoprecipitated DNA (Y axis) was calculated relative to the amount of input DNA. (A) EAST regulates the binding of insulator proteins to Su(Hw)-CP190 sites in S2 cells. Quantitative PCR (qPCR) was performed on the six intergenic and promoter regions bound to by Su(Hw) and CP190 proteins but not by Mod(mdg4)-67.2. Primers were positioned in the middle of the binding region identified in ModEncode by ChIP-seq (S9A Fig). Averaged values of two biological replicates are shown, error bars indicate standard deviations. (B) EAST does not influence the binding of insulator proteins to Su(Hw)-alone sites in S2 cells. Quantitative PCR (qPCR) was performed on the four intergenic and promoter regions bound by Su(Hw) protein alone, in the absence of Mod(mdg4)-67.2 and CP190 proteins. Primers were positioned in the middle of the binding region identified in ModEncode by ChIP-seq (S9B Fig). Error bars indicate standard deviation of two independent biological replicates.
(TIF)

S12 Fig Analysis of insulator proteins binding to chromatin after RNAi knockdown of CP190 in S2 cells. (A) ChIP was performed with antibodies against Su(Hw), Mod(mdg4)-67.2 (the C-terminal region that corresponds to the specific isoform), and Mod-com (the region common to all Mod(mdg4) isoforms) in normal S2 cells (+) and S2 cells after knockdown of CP190 (RNAi CP190). Quantitative PCR (qPCR) was performed on five Su(Hw)-depended insulator sites. Primers were positioned in the middle of the binding region identified in ModEncode by ChIP-seq (S7 Fig). The *ras64B* coding region (Ras) was used as a control devoid of Su(Hw) binding sites. The percent recovery of immunoprecipitated DNA (Y axis) was calculated relative to the amount of input DNA. Averaged values of two biological replicates are shown, error bars indicate standard deviations. (B) Results of ChIP with antibodies against the region common to all Mod(mdg4) isoforms (Mod-com) and dCTCF in normal S2 cells (+) and in S2 cells after knockdown of CP190. Quantitative qPCR was performed on promoter regions of eight genes bound to by dCTCF and CP190 and on the 1A2 insulator. Primers were positioned in the middle of the binding region identified in ModEncode by ChIP-seq. Error bars indicate standard deviation of three independent biological replicates.
(TIF)

S1 Table Summary of yeast two-hybrid analysis of EAST domains for interaction with Su(Hw), Mod(mdg4)-67.2 and CP190. (PDF)

S2 Table Summary of yeast two-hybrid analysis of Mod(mdg4)-67.2 or CP190 domains for interaction with EAST^933-2362^. (PDF)

S3 Table Primer sequences used in PCR for ChIP analysis. (PDF)

S4 Table Primer sequences used in real-time PCR analysis (PDF)
