# Supplementary figures and images for: EAST Organizes Drosophila Insulator Proteins in the Interchromosomal Nuclear Compartment and Modulates CP190 Binding to Chromatin
Source: PLoS One. 2015 Oct 21;10(10):e0140991. doi: 10.1371/journal.pone.0140991 (PMC4638101; doi:10.1371/journal.pone.0140991)

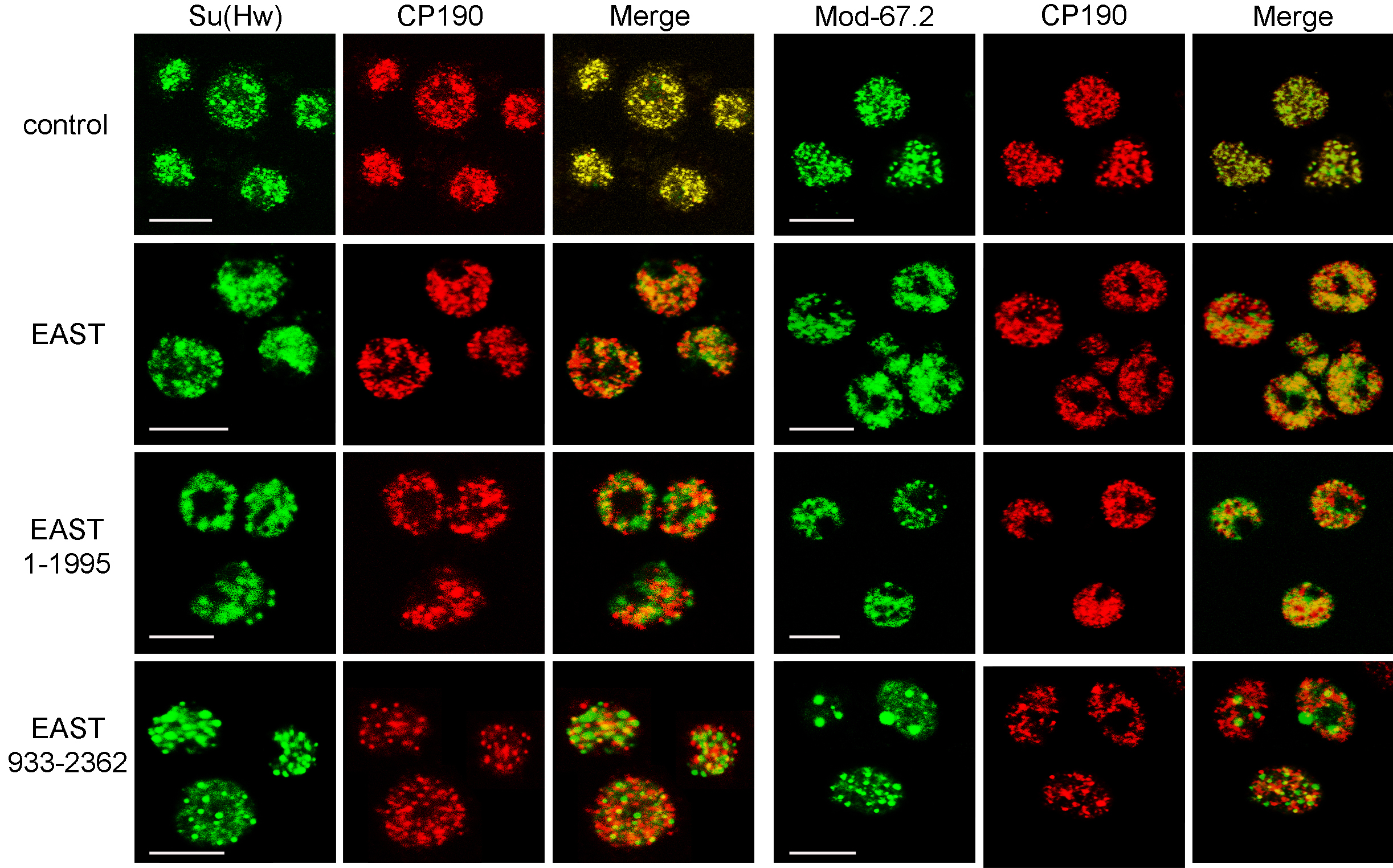

Supplement: S1 Fig — S2 cells was transfected full-length EAST, EAST1-1995, or EAST933-2362 tagged with FLAG×3. Immunostaining with antibodies to Su(Hw) (green), Mod(mdg4)-67.2 (Mod-67.2, green) and CP190 (red). Almost 100% of inspected nuclei show impairment of CP190 co-localization with insulator proteins in speckles. Scale bars, 5 μm. (TIF) [file pone.0140991.s001.tif]

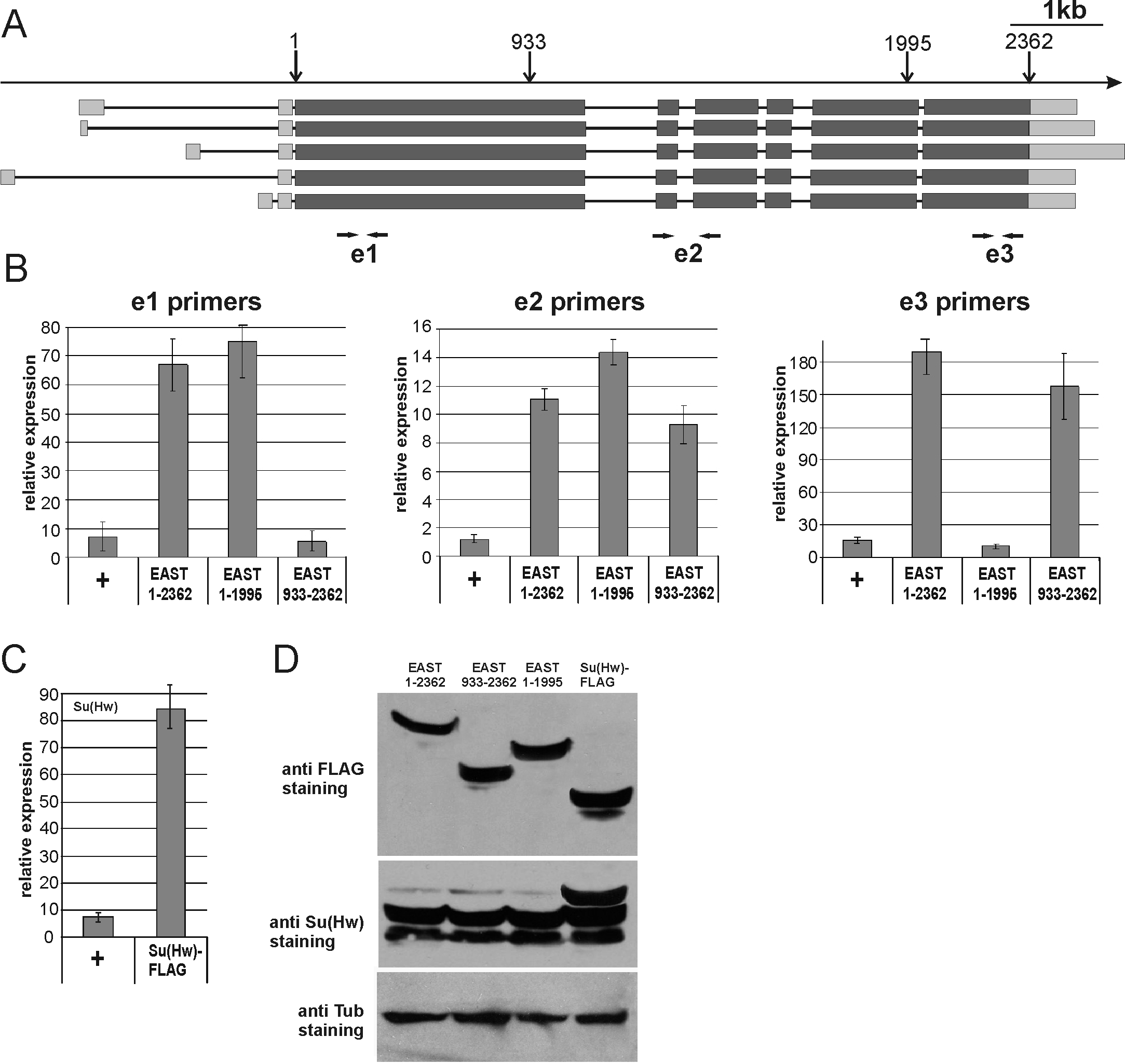

Supplement: S2 Fig — (A) Diagram of lesions in the east locus and alternative east transcripts. Coding regions and UTRs are shown as black and gray rectangles, respectively; the direction of east transcription, by an arrowhead; e1, e2, and e3 are pairs of primers used in qRT-PCR. The numbers of amino acid residues included in the corresponding protein product are shown above the diagram. (B) Expression of the east cDNA and its truncated derivatives. Numbers refer to amino acid residues included in corresponding protein products. RNAs were extracted from normal (+) or transfected S2 cells and quantified by RT-qPCR using appropriate primers. mRNA levels were normalized relative to the ras64B, β-Tubulin56D, and RpL32 gene expression levels, which remain unchanged in S2 cells transfected with the east expression vectors. The experiments were performed on two samples involving independent transfections with a full-length EAST or its fragments, RNA preparations, and RNA reverse transcription into cDNA. Error bars indicate standard deviation of two independent biological replicates. (C) Expression of the Su(Hw)-FLAG cDNA. RNAs were extracted from normal (+) or transfected S2 cells and quantified by RT-qPCR using primers Su(Hw) RT fw /Su(Hw) RT rev (S4 Table). mRNA levels were normalized relative to the ras64B, β-Tubulin56D, and RpL32 gene expression levels, which remain unchanged in S2 cells transfected with the Su(Hw)-FLAG expression vectors. (D) Expression of Su(Hw) protein in S2 cells transfected with EAST-FLAG derivatives. In the top panel, cotransfected EAST-FLAG derivatives and Su(Hw)-FLAG are probed with anti FLAG antibodies. And in bottom panel, the same membrane was probed with anti Su(Hw) antibodies. In the bottom panel we can see that the level of wild type Su(Hw) protein expression is relatively equal to the transfected Su(Hw)-FLAG protein and the presence of EAST expressed derivatives doesn’t significantly influence Su(Hw) expression. Staining with anti-Tubulin (Tub) antibodies (b [file pone.0140991.s002.tif]

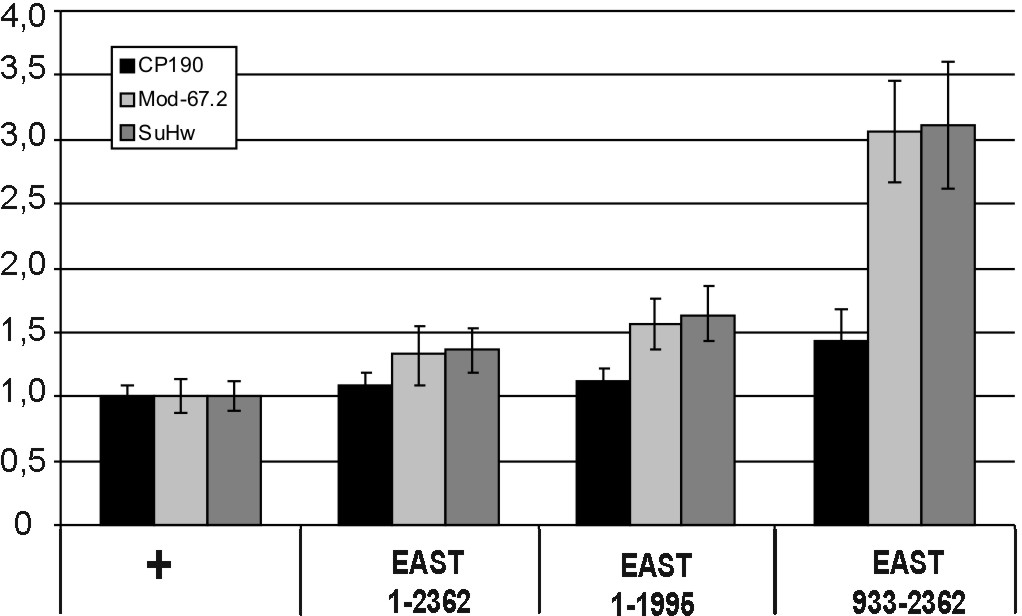

Supplement: S3 Fig — Using scale bar as a reference we measured in five nuclei at least twenty speckles diameters. Non transfected nuclei were used as control. Speckles enlargement in cells transfected with full length EAST (1–2362), EAST 1–1995 or EAST 933–2362 represent relatively to the non transfected cells (+). (TIF) [file pone.0140991.s003.tif]

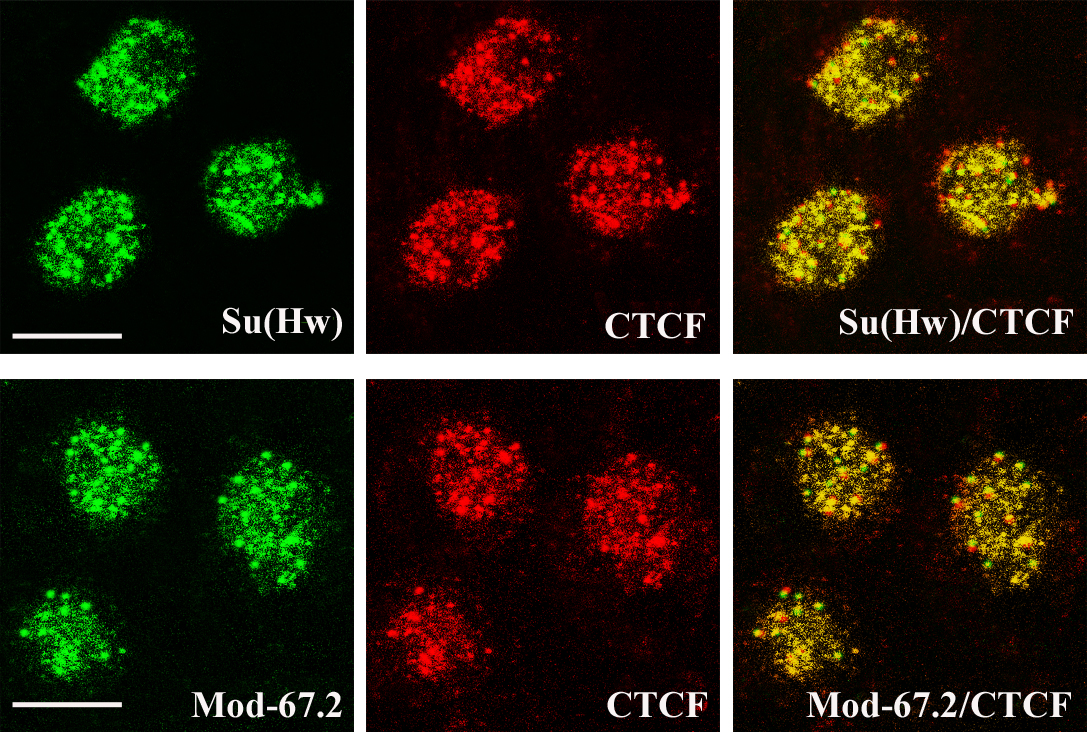

Supplement: S4 Fig — Immunostaining with antibodies to Su(Hw) (green), Mod(mdg4)-67.2 (Mod-67.2, green) and dCTCF (red). This staining was done using distinct (N-terminal region) anti-dCTCF (CTCF) antibodies. Scale bars, 5 μm. (TIF) [file pone.0140991.s004.tif]

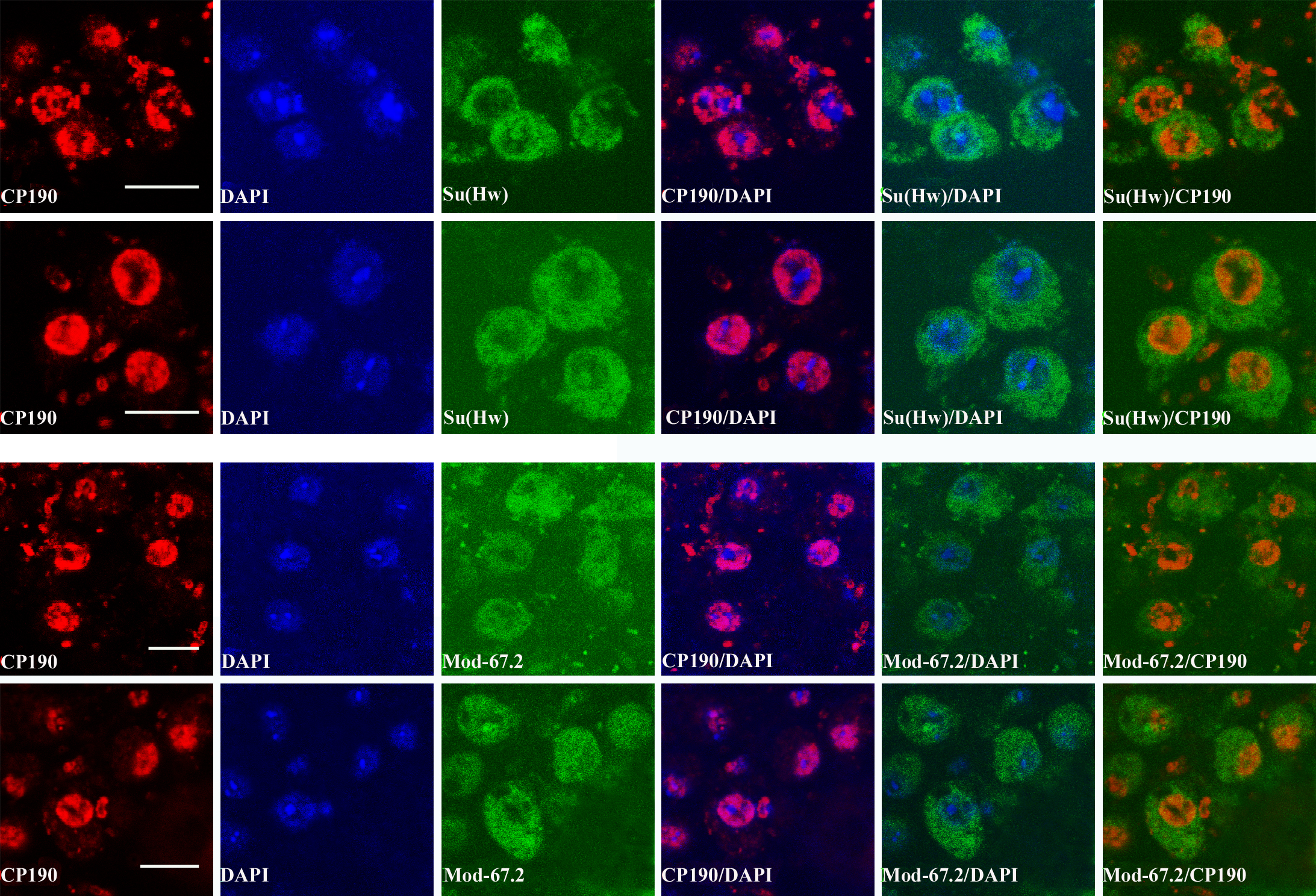

Supplement: S5 Fig — Immunostaining with antibodies to CP190 (red), SuHw (green), and Mod(mdg4)-67.2 (green). DAPI staining (blue) was used to visualize the nuclei. Scale bars, 5 μm. (TIF) [file pone.0140991.s005.tif]

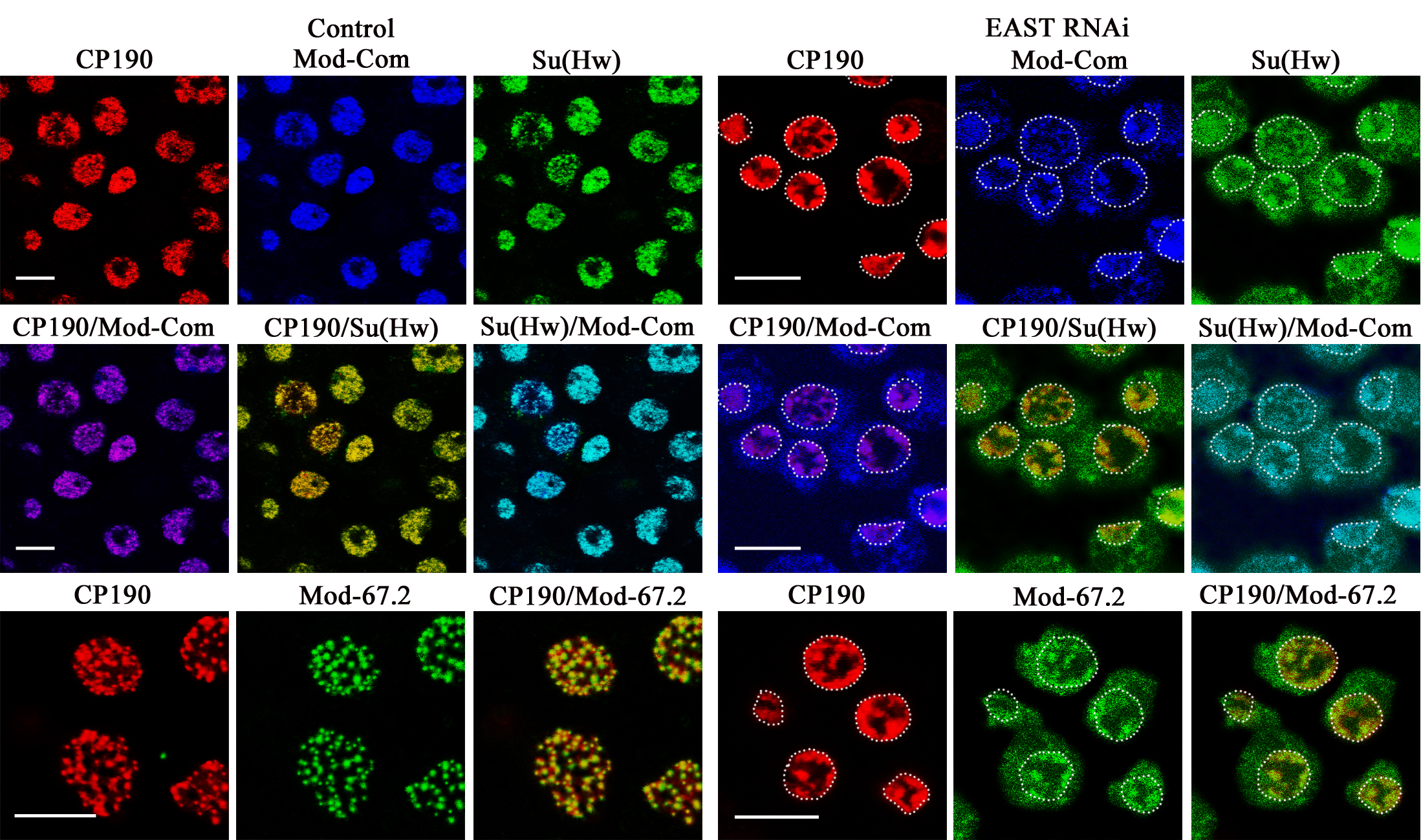

Supplement: S6 Fig — Nontransfected S2 cells designated as «Control» and S2 cells after EAST knockdown by RNAi designated as «EAST RNAi». Immunostaining with antibodies to CP190 (red), Su(Hw) (green), Mod(mdg4)-67.2 (Mod-67.2, green), and common part of Mod(mdg4) (Mod-Com, blue). Dotted lines indicate the nucleus boundaries (See S5 Fig). Scale bars, 5 μm. The second combination of primers (5'—gaaacccagaatgacaggtgggat- 3' and 5'—gctgttactgttggctccttag- 3') was generated for the 5’ end of east transcript. (TIF) [file pone.0140991.s006.tif]

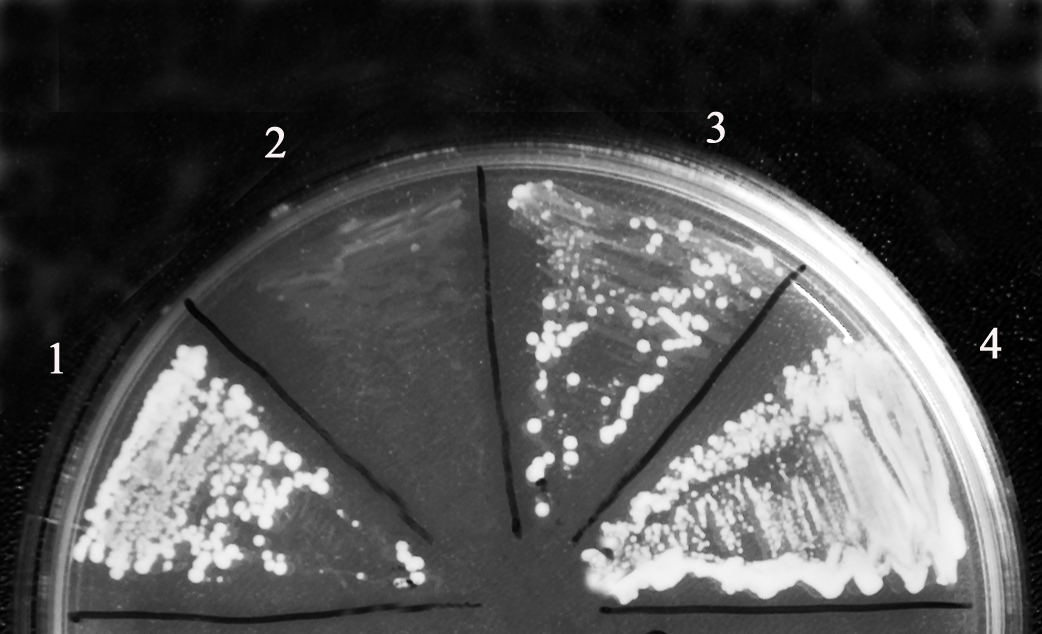

Supplement: S7 Fig — (TIF) [file pone.0140991.s007.tif]

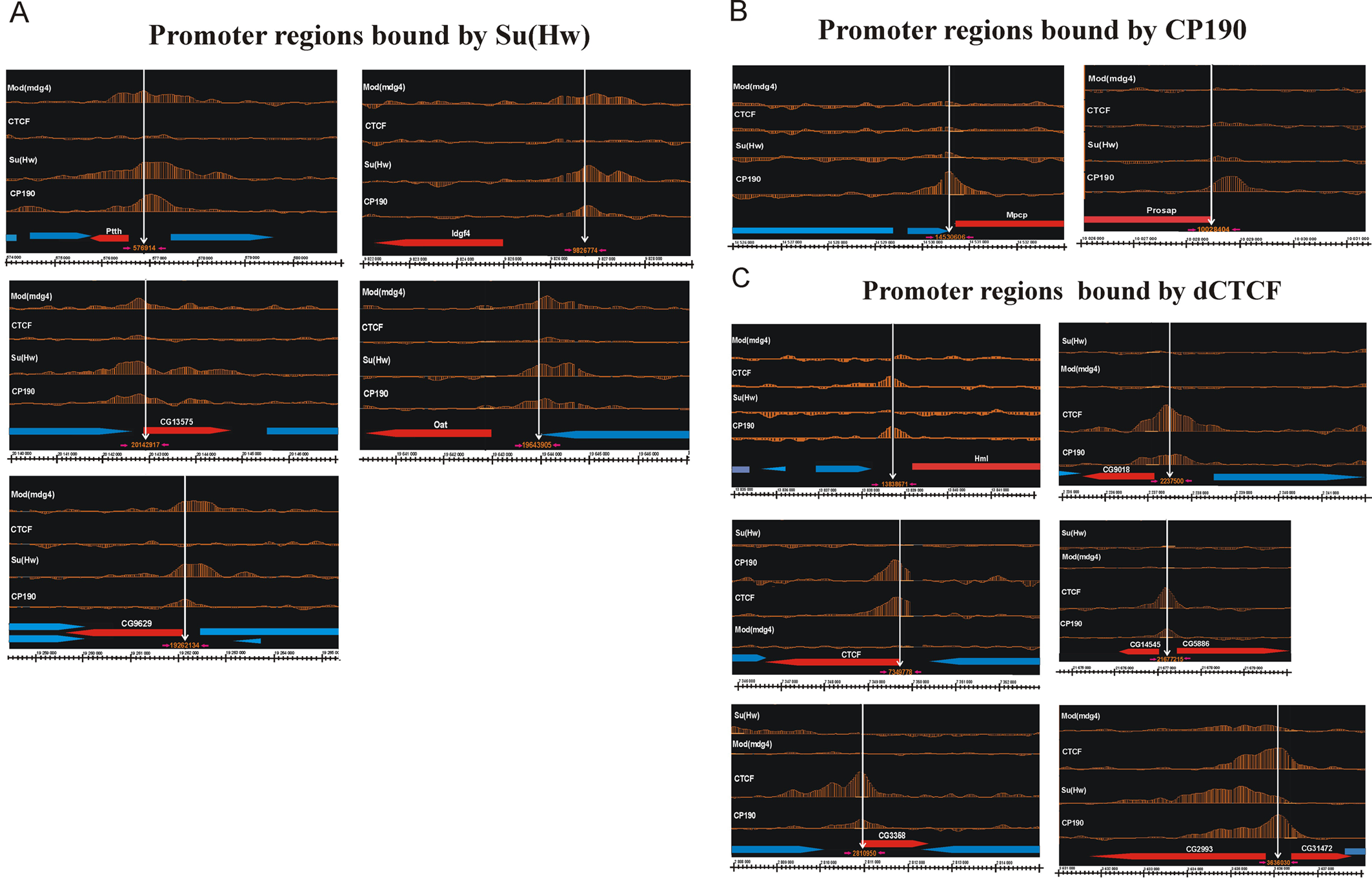

Supplement: S8 Fig — Genes examined for expression are marked red. The regions used in qPCR are indicated by vertical white arrows and numbered according to their chromosome position (FlyBase, 2006). (TIF) [file pone.0140991.s008.tif]

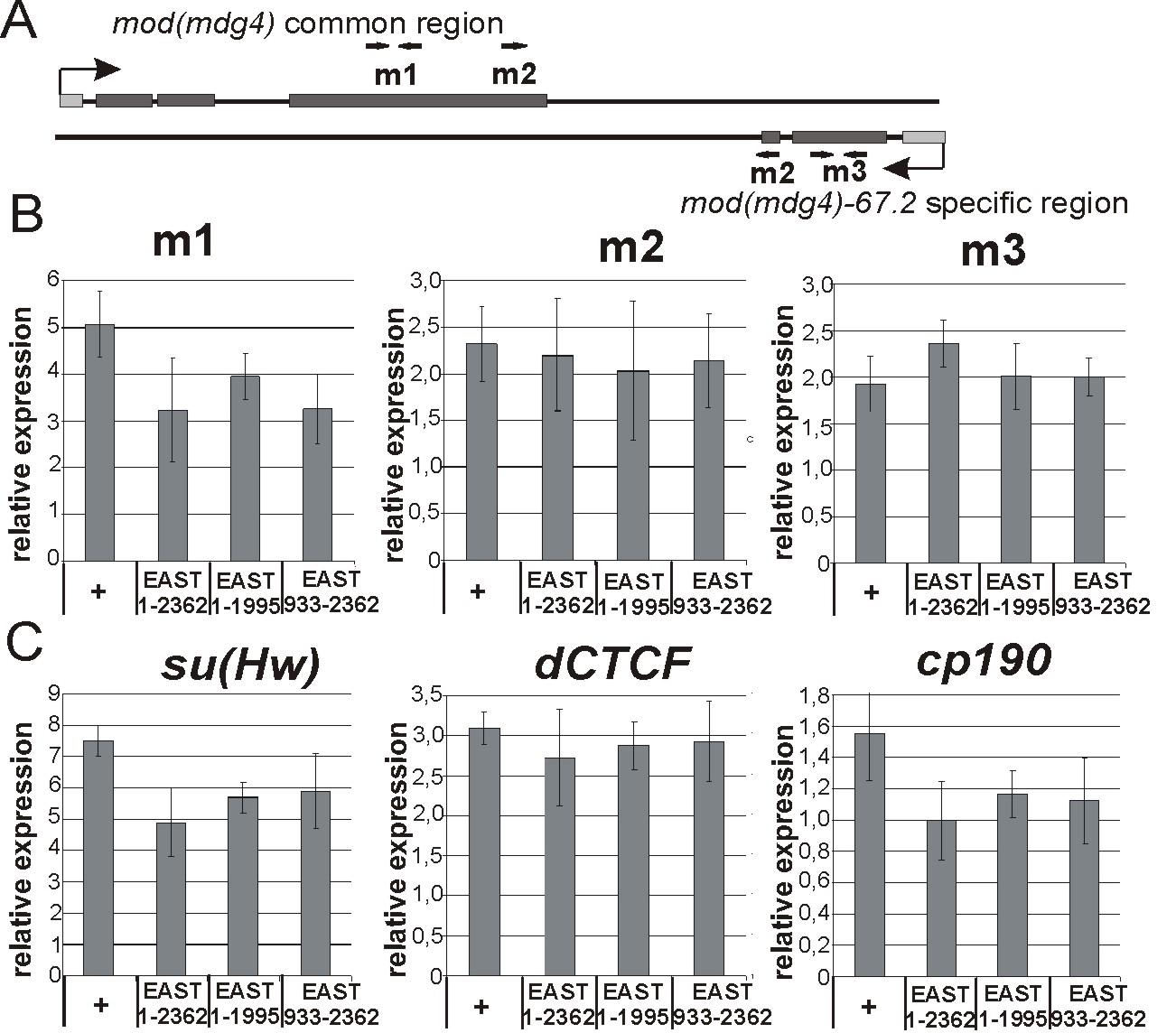

Supplement: S9 Fig — (A) Scheme of the mod(mdg4) gene encoding the Mod(mdg4)-67.2 isoform. The mRNA for the Mod(mdg4)-67.2 protein is formed by trans-splicing of two RNAs encoded by genes located on the opposite DNA strands. Positions of primer pairs used in qRT-PCR (m1, m2, and m3) are indicated by arrows. (B) Expression of the gene encoding Mod(mdg4)-67.2 in normal S2 cells (+) and in transfected S2 cells expressing FLAG×3-tagged EAST1-2362, EAST1-1995 or EAST933-2362. (C) Expression of su(Hw), DCTCF and cp190 genes in S2 cells transfected with EAST variants. mRNA levels were normalized relative to the level of ras64B, β-Tubulin56D, and RpL32 gene expression, which remains unchanged in S2 cells transfected with the east expression vectors. The experiments were performed on two samples involving independent transfections with a full-length EAST or its fragments, RNA preparations, and RNA reverse transcription into cDNA. Error bars indicate standard deviation of two independent biological replicates. (TIF) [file pone.0140991.s009.tif]

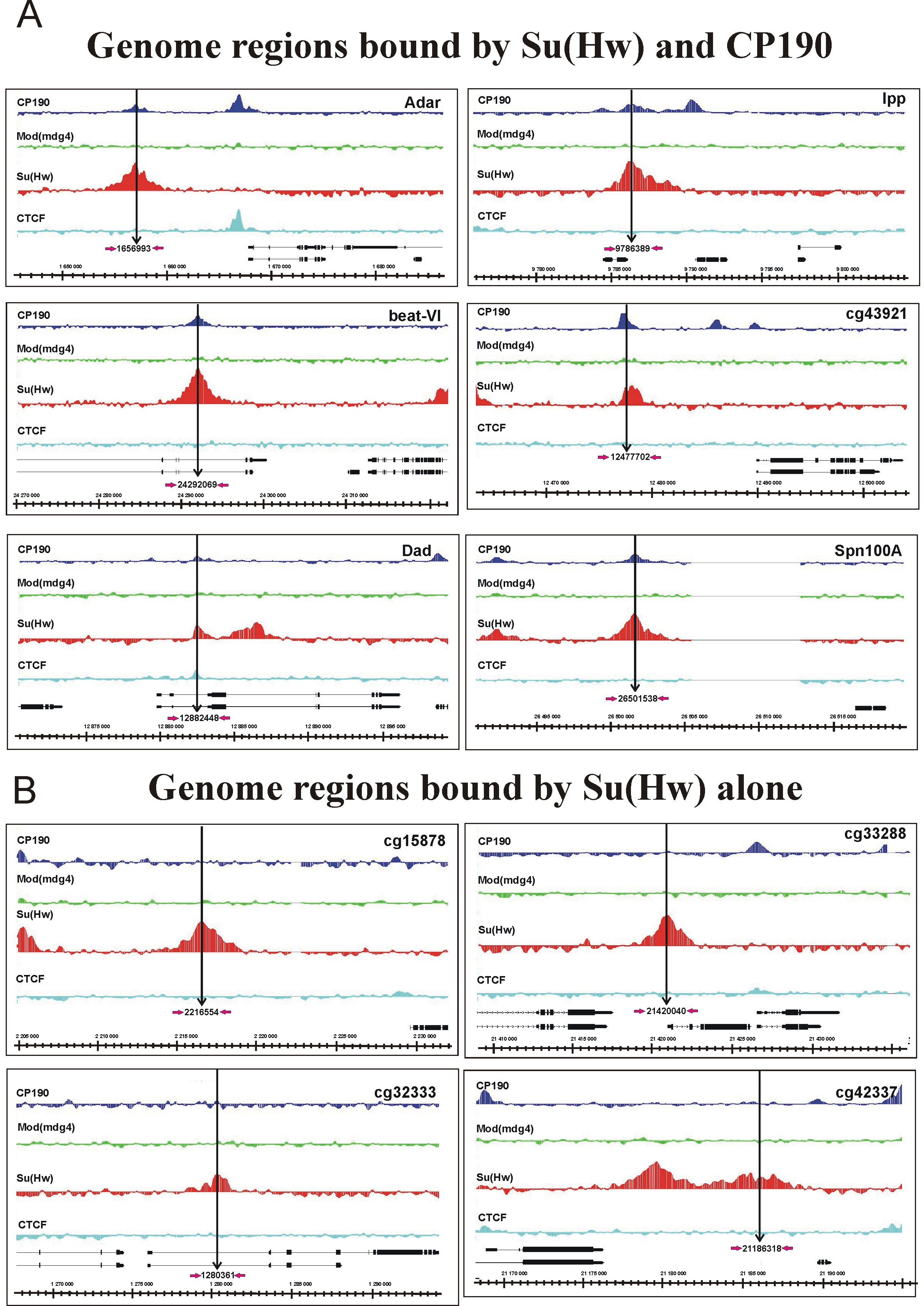

Supplement: S10 Fig — The regions used in qPCR are indicated by vertical black arrows and numbered according to their chromosome position (FlyBase, 2006). Names of the identified genomic regions are given in the upper right corner of pictures. (TIF) [file pone.0140991.s010.tif]

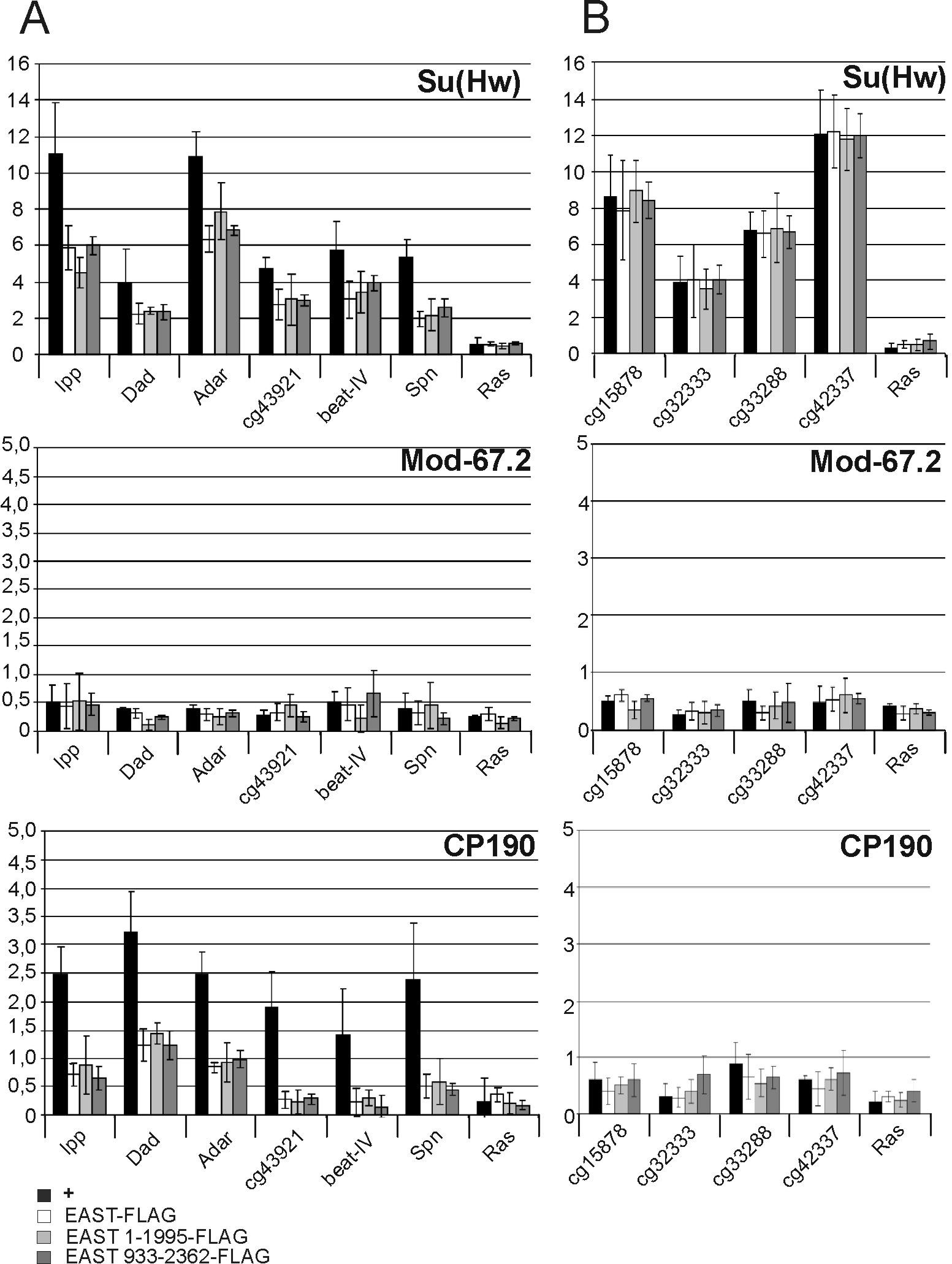

Supplement: S11 Fig — ChIP was performed with antibodies against Su(Hw), CP190 and Mod(mdg4)-67.2 (the C-terminal region that corresponds to the specific isoform) in normal S2 cells (+) and in transfected S2 cells expressing FLAG×3-tagged EAST, EAST1-1995, or EAST933-2362. The ras64B coding region (Ras) was used as a control devoid of Su(Hw) binding sites. The percent recovery of immunoprecipitated DNA (Y axis) was calculated relative to the amount of input DNA. (A) EAST regulates the binding of insulator proteins to Su(Hw)-CP190 sites in S2 cells. Quantitative PCR (qPCR) was performed on the six intergenic and promoter regions bound to by Su(Hw) and CP190 proteins but not by Mod(mdg4)-67.2. Primers were positioned in the middle of the binding region identified in ModEncode by ChIP-seq (S9A Fig). Averaged values of two biological replicates are shown, error bars indicate standard deviations. (B) EAST does not influence the binding of insulator proteins to Su(Hw)-alone sites in S2 cells. Quantitative PCR (qPCR) was performed on the four intergenic and promoter regions bound by Su(Hw) protein alone, in the absence of Mod(mdg4)-67.2 and CP190 proteins. Primers were positioned in the middle of the binding region identified in ModEncode by ChIP-seq (S9B Fig). Error bars indicate standard deviation of two independent biological replicates. (TIF) [file pone.0140991.s011.tif]

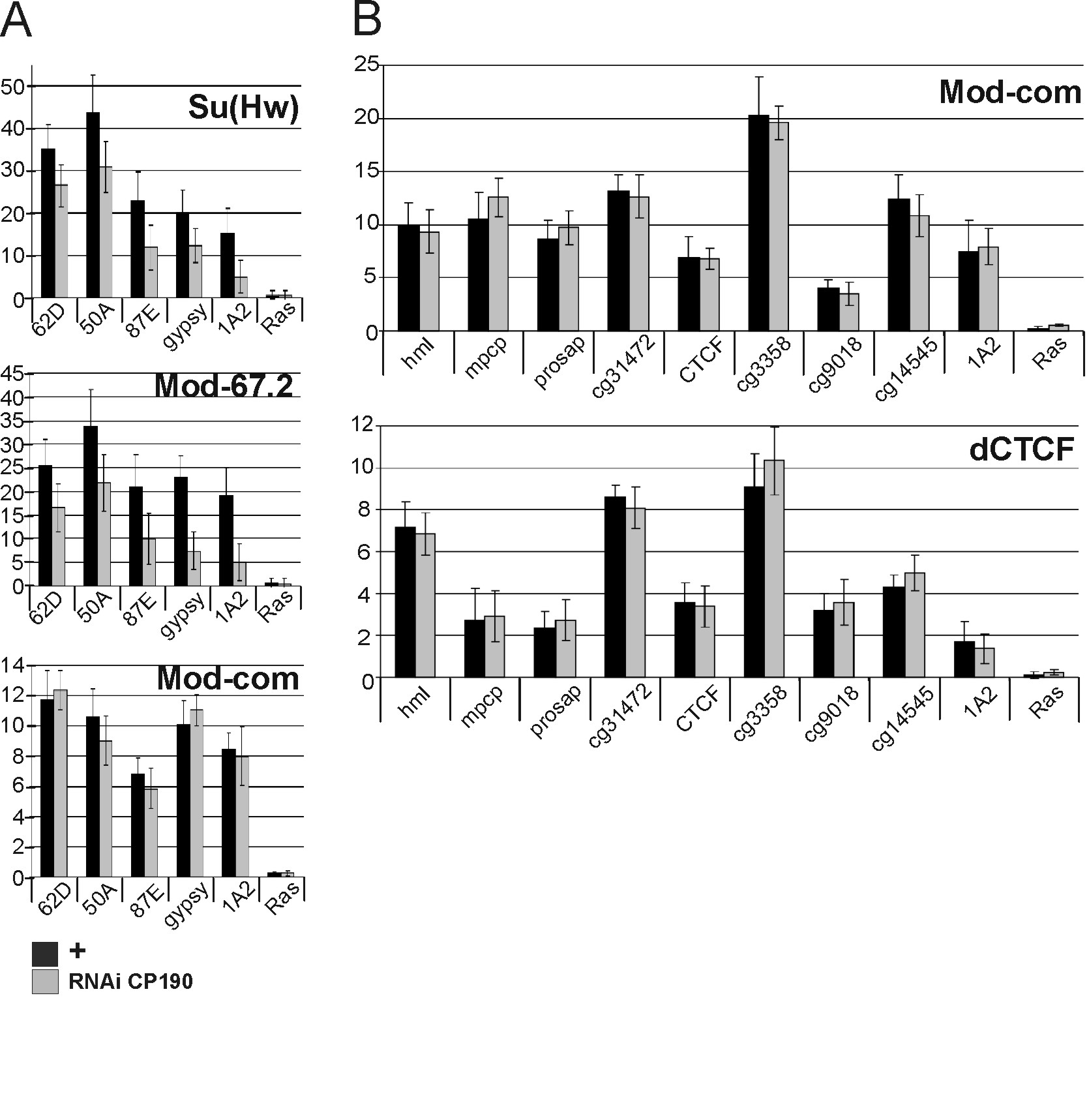

Supplement: S12 Fig — (A) ChIP was performed with antibodies against Su(Hw), Mod(mdg4)-67.2 (the C-terminal region that corresponds to the specific isoform), and Mod-com (the region common to all Mod(mdg4) isoforms) in normal S2 cells (+) and S2 cells after knockdown of CP190 (RNAi CP190). Quantitative PCR (qPCR) was performed on five Su(Hw)-depended insulator sites. Primers were positioned in the middle of the binding region identified in ModEncode by ChIP-seq (S7 Fig). The ras64B coding region (Ras) was used as a control devoid of Su(Hw) binding sites. The percent recovery of immunoprecipitated DNA (Y axis) was calculated relative to the amount of input DNA. Averaged values of two biological replicates are shown, error bars indicate standard deviations. (B) Results of ChIP with antibodies against the region common to all Mod(mdg4) isoforms (Mod-com) and dCTCF in normal S2 cells (+) and in S2 cells after knockdown of CP190. Quantitative qPCR was performed on promoter regions of eight genes bound to by dCTCF and CP190 and on the 1A2 insulator. Primers were positioned in the middle of the binding region identified in ModEncode by ChIP-seq. Error bars indicate standard deviation of three independent biological replicates. (TIF) [file pone.0140991.s012.tif]
