# Supplementary material for: EAST Organizes Drosophila Insulator Proteins in the Interchromosomal Nuclear Compartment and Modulates CP190 Binding to Chromatin
Source: PLoS One. 2015 Oct 21;10(10):e0140991. doi: 10.1371/journal.pone.0140991 (PMC4638101; doi:10.1371/journal.pone.0140991)
Supplement: S1 Table — (PDF) [file pone.0140991.s013.pdf]

S1 Table. Summary of yeast two-hybrid analysis of EAST domains for interaction with Su(Hw), Mod(mdg4)-67.2 and CP190

	**EAST bait**					**EAST prey**				
	**1–933**	**933–1995**	**1995–2362**	**1–1995**	**933–2365**	**1–933**	**933–1995**	**1995–2362**	**1–1995**	**933–2365**
**Su(Hw)**	–	–	–	–	–	–	–	–	–	–
**Mod-67.2**	–	+++	++	++	+++	–	+++	++	+	+++
**CP190**	–	+++	–	+	+++	–	+++	–	+	+++

Two-hybrid assays were carried out using yeast strain pJ694A, plasmids, and protocols from Clontech. For growth assays, plasmids were transformed into yeast strain pJ694A by the lithium acetate method, as recommended by the manufacturer, and plated onto media without tryptophan and leucine. After two days of growth at 30°C, the cells were plated onto selective media without tryptophan, leucine, histidine, and adenine, and characteristics of their growth were compared. No growth was observed after transformation with single plasmids, indicating that interactions between the proteins are required for the expression of the reporter genes (data not shown). The (+) signs indicate the relative strength of the two-hybrid interaction, and the (–) sign indicates the absence of interaction.
