# Supplementary material for: EAST Organizes Drosophila Insulator Proteins in the Interchromosomal Nuclear Compartment and Modulates CP190 Binding to Chromatin
Source: PLoS One. 2015 Oct 21;10(10):e0140991. doi: 10.1371/journal.pone.0140991 (PMC4638101; doi:10.1371/journal.pone.0140991)
Supplement: S2 Table — (PDF) [file pone.0140991.s014.pdf]

S2 Table. Summary of yeast two-hybrid analysis of Mod(mdg4)-67.2 or CP190 domains for interaction with EAST$^{933\text{-}2362}$

Mod(mdg4)-67.2	EAST$^{933\text{-}2362}$ bait	EAST$^{933\text{-}2362}$ prey
1–610	+++	+++
1–510	+++	++
1–465	+++	++
1–273	+++	+++
120–610	–	–
1–610 (Δ145–273)	+++	++
1–610 (Δ310–390)	+++	++
1–145	+++	+++

CP190	EAST$^{933\text{-}2362}$ bait	EAST$^{933\text{-}2362}$ prey
1–1097	+++	+++
1–767	+++	+++
1–610	+++	+++
125–610	–	–
1–542	++	++
1–308	–	–
1–1097 (308–430)	+	–

Designations are as in Table S1.
