# Supplementary material for: EAST Organizes Drosophila Insulator Proteins in the Interchromosomal Nuclear Compartment and Modulates CP190 Binding to Chromatin
Source: PLoS One. 2015 Oct 21;10(10):e0140991. doi: 10.1371/journal.pone.0140991 (PMC4638101; doi:10.1371/journal.pone.0140991)
Supplement: S3 Table — (PDF) [file pone.0140991.s015.pdf]

S3 Table. Primer sequences used in PCR for ChIP analysis

Primer pair	Sequences
Idgf4 fw<br>Idgf4 rev	5' AAGGGCACTTCAGTTCACGATT 3'<br>5' CACTATTCGAGCATTGCATTCG 3'
ptth fw<br>ptth rev	5' GTGCAAAGGCAGATGAATGGTA 3'<br>5' GCCTCTTGGACAGTCGTTCT 3'
cg9629 fw<br>cg9629 rev	5' GGCTTTTGTGGGCAGCTTATC 3'<br>5' TGCGATGGATCCTAGGGAAATG 3'
hlm fw<br>hml rev	5' CGCATGAACCGTAAACTGCTTG 3'<br>5' CTTCTCCAATGGCATTGAACTTG 3'
cg31472 fw<br>cg31472 rev	5' CACAAGGTGGGACTGGCTAC 3'<br>5' CTTTTCTGCGGTACTTGAGCAC 3'
Mpcp fw<br>Mpcp rev	5' CTCACCTGATACCTCGTATGC 3'<br>5' GTTTTGCCGTGGAGAGGTGTA 3'
cg13575 fw<br>cg13575 rev	5'GGAACGACGTGCGCATAGTAA 3'<br>5' GACAGATTTATGGCCAGCGACT 3'
prosap fw<br>prosap rev	5' CGTTGGGCTTACAGCTCTAG 3'<br>5' GCGCAATCCGTCATACAGAAC 3'
Oat fw<br>Oat rev	5' CGCGTTTCATTAGCTACCTCGAA 3'<br>5' GAAATGGCAGTGGGATCGGAAT 3'
dCTCF fw<br>dCTCF rev	5' TGCGTCCACATGAAACAGCTG 3'<br>5'TCCTTGGCTTATCCAGTATCCA 3'
cg9018 fw<br>cg9018 rev	5' GGCTGTGTCATCAATCAAGTG 3'<br>5' CTCCAACGAGCCCACCACA 3'
cg3358 fw<br>cg3358 rev	5' GCTGGCACGTCTGAGCTTAG 3'<br>5' CAGCTGTTTCACTTGCGAGATT 3'
cg14545 f<br>cg14545 rev	5' TGCAGCACCTGGCGGTAGATT 3'<br>5' CGCATCCAACTGGTCACACT 3'
62D fw<br>62D rev	5' TTTGGGCTTGGTGAGAACAG 3'<br>5' TGATACCAGGCGAACAGAAATC 3'
50A fw<br>50A rev	5' ATACAAAGTGGTTTCAGCCAAGAAG 3'<br>5' TTGATAAATAGTCCAGCACGCATAC 3'
87E fw<br>87E rev	5' GGATGTTACA TTGAGAGTGCTTAGG3'<br>5' TTTGCGTTTCGGCTGCTGTC 3'

1A2 fw<br>1A2 rev	5' ACCACACATCAGTCATCGTGT 3'<br>5' CTTCGTCTACCGTTGTGC 3'
Gypsy fw<br>Gypsy rev	5' TTCTCTAAAAAGTATGCAGCACTT 3'<br>5'CACGTAATAAGTGTGCGTTGA 3'
Ras fw<br>Ras rev	5' GAGGGATTCCTGCTCGTCTTCG 3'<br>5' GTCGCACTTGTTACCCACCATC 3'
42337 fw<br>42337 rev	5' TTGATGTCCCTCCGCTCTCA 3'<br>5' CCAACATTGACCATCCAACGAG 3'
15878 fw<br>15878 rev	5' GTGAACTCAATTTGCTGGCATC 3'<br>5' CGTTGTGTTCGCCGCTTTTG 3'
cg32333 fw<br>cg32333 rev	5' CAAAAAGTTGCCGGACGACCAAA 3'<br>5' CTACTTGCCAGCGCCAACCTT 3'
cg33288 fw<br>cg33288 rev	5' CATGGCTCGTATTCCGAGAGAT 3'<br>5' CTATGGAAATGTTAGCGAGTGAG 3'
beat fw<br>beat rev	5' AATCAGCCGTTAACCGCTAAAG 3'<br>5' CATGGCATCATCATCATCAGTAG 3'
Dad fw<br>Dad rev	5' TCGTCGATAAGGAGCGCAAGA 3'<br>5' CGACGCCAGTGCATATGCAA 3'
Ipp fw<br>Ipp rev	5' GGATTATAGCTGCTGTTTGGAAG 3'<br>5' TACCGCACAGAAACTCCAATAAG 3'
Spn fw<br>Spn rev	5' AGTGTTGCGGATTGCCTGCAT 3'<br>5' AGTACGTGCTGCGGCGACAA 3'
Adar fw<br>Adar rev	5' CTAGCAAGCCGATGATGAAGTTG 3'<br>5' GGTAAGGTTCACGCGAACCTAA 3'
43921 fw<br>43921 rev	5' CAGGACGATTGCGCAGCAAAG 3'<br>5' GCCCGCTATTGGTGGTGTTC 3'
