# Supplementary material for: EAST Organizes Drosophila Insulator Proteins in the Interchromosomal Nuclear Compartment and Modulates CP190 Binding to Chromatin
Source: PLoS One. 2015 Oct 21;10(10):e0140991. doi: 10.1371/journal.pone.0140991 (PMC4638101; doi:10.1371/journal.pone.0140991)
Supplement: S4 Table — (PDF) [file pone.0140991.s016.pdf]

S4 Table. Primer sequences used in real-time PCR analysis

Primer pair	Sequences
Idgf4 RT fw<br>Idgf4 RT rev	5' ACGGCAACAGTTTTGTCCGCG 3'<br>5' TTGTTCTCTGGATCCACGGTG 3'
ptth RT fw<br>ptth RT rev	5' CTCGTCCAAACCTTCATCGCT 3'<br>5' GGCACAGATCGAGCATGATG 3'
cg9629 RT fw<br>cg9629 RT rev	5' CCGGTGAAGGAAACGAGGTTA 3'<br>5' GTGCTGCGTAGGAACAATCTG 3'
hml RT fw<br>hml RT rev	5' CAAGGGCATTAAACTGGAGCAC 3'<br>5' CTCCTTGTACTGCACAGTTCC 3'
cg31472 RT fw<br>cg31472 RT rev	5' CAATTCGAAGGATGAGCCAAGG 3'<br>5' TCCAGCCAGTCTCGGAATAC 3'
Mpcp RT fw<br>Mpcp RT rev	5' AGTTCGCTTGCTTCGAGCGCA 3'<br>5' CCGTTCCACATGCCACTGAAG 3'
cg13575 RT fw<br>cg13575 RT rev	5' TCTCCTGGATGTGGAGGACT 3'<br>5' GCAGGAAGATCAGGGTGAAC 3'
prosap RT fw<br>prosap RT rev	5' GATGTCAAGCAGCAGGTGCT 3'<br>5' CCGTTGGCAGGTGGAGCAAA 3'
Oat RT fw<br>Oat RT rev	5' CTTCTCCAGGTAGCCCCACT 3'<br>5' CCTCCAAGTTGGCCTTGACAT 3'
dCTCF RT fw<br>dCTCF RT rev	5' CGGGCTGGTCGAACGCTAA 3'<br>5' ACGTTCCGTGGTCAGTACAG 3'
cg9018 RT fw<br>cg9018 RT rev	5' GCATTGTAGTCGTTCAGCAGAT3'<br>5'AGGACAAGGCCAAGGAACTG 3'
cg3358 RT fw<br>cg3358 RT rev	5'GTGGATGAGGCACTGGAACT 3'<br>5' GATCGCAACTGGTCATAGTAG 3'
cg14545 RT fw<br>cg14545 RT rev	5'GAGCGATGACATCGGTGTACA 3'<br>5' ACGAAGTGGAGGAGTATGTGG 3'
rpl32 RT fw<br>rpl32 RT rev	5' GTTCGATCCGTAACCGATGTTG 3'<br>5' CCAGTCGGATCGATATGCTAAG 3'
cg7943 RT fw<br>cg7943 RT rev	5' GACTCCAAGTTCCACCAGCA 3'<br>5' GCCTCTTCTCGCAGTACGA 3'
cg2993 RT fw<br>cg2993 RT rev	5' ATCCAGCGTATCTTGGCATAGC 3'<br>5' TGCCCATTGAGTGCCTTATATC 3'

cg10433 RT fw<br>cg10433 RT rev	5' CACAATCCCTTGTTCGAGGTG 3'<br>5' CAAACGATGTGAATGACCCCAGT 3'
cg17090 RT fw<br>cg17090 RT rev	5' CCTAAGGCAACCTGCAACATC 3'<br>5' GCCGTGGCTGGAGTTATATG 3'
Cyp9f2 RT fw<br>Cyp9f2 RT rev	5' AGCCAGTGCTCTACTTTGGCA 3'<br>5' CATCGCTGCTGGTGGCAAACA 3'
Idgf2 RT fw<br>Idgf2 RT rev	5' GCCTCCAACTTAGTCTGCTAC 3'<br>5' GAGGGAGGTAACCTCGGAGAA 3'
East e1RT fw<br>East e1RT rev	5' CTATAGCACCAGTGACACCCACTTG 3'<br>5' TTGCTCTGTTACTGAGGAGGATGCA 3'
East e2 RT fw<br>East e2 RT rev	5' GCGGTTTCTCCTCGTCGTCATCCT 3'<br>5' CAAGGAGAACTTGTCCACGTCTG 3'
East e3 RT fw<br>East e3 RT rev	5' ATGCCTTAGAACTGCCTCTGTGGAC 3'<br>5' TGGACTGGTGCGACTGCTGTAAC 3'
Mod e1 RT fw<br>Mod e1 RT rev	5' CTACGACGACAGCTACTTCAC 3'<br>5' AGCCTCTGTGTTGCCCTGATC 3'
Mod e2 RT fw<br>Mod e2 RT rev	5' CTACGACGACAGCTACTTCAC 3'<br>5' CTGGGCGACAGCGAGGATATGA 3'
Mod e3 RT fw<br>Mod e3 RT rev	5' TGAGCACGGCGAGGAGTGCAA 3'<br>5' CTGGGCGACAGCGAGGATATGA 3'
Su(Hw) RT fw<br>Su(Hw) RT rev	5' ATCGAGGGAGAGCAGCTGACGGAT 3'<br>5' GCCACTGAAGTCATCCTCGCTACA 3'
CP190 RT fw<br>CP190 RT rev	5' TCCAGTCTCTACACAAGGGACCCG 3'<br>5' TTCTCGTCCGCCGCTTCTGAGT 3'
Ras RT fw<br>Ras RT rev	5' GAGGGATTCCTGCTCGTCTTCG 3'<br>5' GTCGCACTTGTTACCCACCATC 3'
eh RT Fw<br>eh RT Rev	5’ AGCATGGTCTTGCACTGGAC 3’<br>5’ GTTGCAATGTGCCTGGTGCAT 3’
Tub RT Fw<br>Tub RT Rev	5' GAGAACACGGACGAGACCTACTG 3'<br>5' GAATCGGAGGCAGGTGGTTACG 3'
